# A Review on Stems Composition and Their Impact on Wine Quality

**DOI:** 10.3390/molecules26051240

**Published:** 2021-02-25

**Authors:** Marie Blackford, Montaine Comby, Liming Zeng, Ágnes Dienes-Nagy, Gilles Bourdin, Fabrice Lorenzini, Benoit Bach

**Affiliations:** 1 Agroscope, Route de Duillier 50, 1260 Nyon, Switzerland; montaine.comby@outlook.fr (M.C.); agnes.dienes-nagy@agroscope.admin.ch (Á.D.-N.); gilles.bourdin@agroscope.admin.ch (G.B.); fabrice.lorenzini@agroscope.admin.ch (F.L.); 2 Changins, Viticulture and Enology, HES-SO University of Applied Sciences and Arts Western Switzerland, Route de Duillier 50, 1260 Nyon, Switzerland; liming.zeng@changins.ch (L.Z.); benoit.bach@changins.ch (B.B.)

**Keywords:** grape stems, whole clusters, wine, winemaking practice, phenolic compounds, antioxidant activity, maceration technique, polyphenolic compounds

## Abstract

Often blamed for bringing green aromas and astringency to wines, the use of stems is also empirically known to improve the aromatic complexity and freshness of some wines. Although applied in different wine-growing regions, stems use remains mainly experimental at a cellar level. Few studies have specifically focused on the compounds extracted from stems during fermentation and maceration and their potential impact on the must and wine matrices. We identified current knowledge on stem chemical composition and inventoried the compounds likely to be released during maceration to consider their theoretical impact. In addition, we investigated existing studies that examined the impact of either single stems or whole clusters on the wine quality. Many parameters influence stems’ effect on the wine, especially grape variety, stem state, how stems are incorporated, when they are added, and contact duration. Other rarely considered factors may also have an impact, including vintage and ripening conditions, which could affect the lignification of the stem.

## 1. Introduction

For white winemaking, stems are generally kept during pressing because they allow for better juice extraction yields. Given the short contact time, compounds are extracted from the stems in relatively low levels. In red winemaking, the maceration phase—where color is extracted from the grape skin and tannins from the grape seeds—occurs before pressing. Originally, stems were kept during this phase, but destemming practices appeared at the end of the 19th century, improving wine quality by reducing excessive astringency and negative strong green tastes from the stems [1]. Initially used in cellars with high production capacity, the first destemming machines quickly trivialized this practice; today, this technique is systematic for winegrowers in most wine-producing countries [2]. However, in some regions, using whole clusters of grapes is a matter of tradition. The stem is considered a natural additive that, if well mastered, brings complexity, freshness, and phenolic structure to the wine and facilitates chemical stability during aging [3] (e.g., the Pinot Noir in Burgundy, the Cabernet Franc in the Loire Valley, or the Gamay in the Beaujolais, Kakhethian wines from Georgia, etc.). In recent years, winemakers in Europe and other countries, such as Australia and South Africa, have shown an interest in using stems, and several technical articles mention the advantages of this practice [4,5].

These winemaking techniques are not used for all grape varieties, nor for all vintages. Since these techniques have very little research behind them, they are generally passed along by word of mouth. Therefore, it is difficult to know which stem conditions will lead to improved or deteriorated wine quality.

Stem composition has often been analyzed to value winemaking by-products and has been relatively well studied. Many compounds of interest can be found in stems’ overall composition. Their richness in polyphenolic compounds makes them very interesting for the food and medicine industries, in relation to their antioxidant potential. In some studies, units used to express stem extract composition is very specific and makes it impossible to compare results. Therefore, such data are not presented in this article [6,7,8,9,10]. This review gathered information from the literature on stem chemical composition to examine how these compounds contribute to variations in aroma and taste when stems are included during winemaking. Although stems are only used for red wines, we also examined data on the chemical composition of white grape variety stems. We then compiled the main results observed when whole clusters of grapes or single stems were incorporated into the winemaking process, from a technological, chemical, and sensorial perspective.

## 2. Grape Stems

### 2.1. Morphology

**Figure 1:**
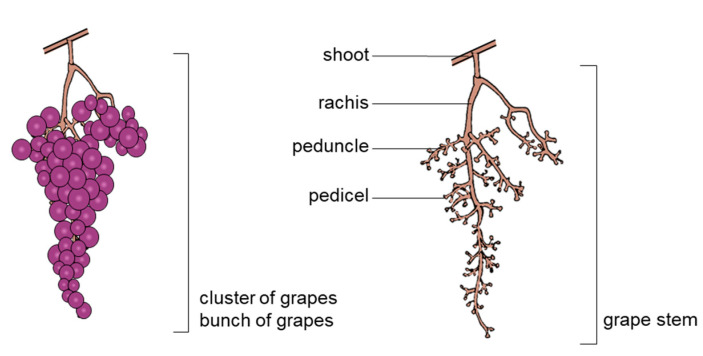
Bunch of grapes and stem morphology.

The stem is the skeleton of the grape cluster or bunch. The longest part, the rachis (main axis), is branched with peduncles, and a pedicel attaches each grape berry to the stem (Figure 1).

The stem’s final size is reached around veraison [1]. For each grape variety, the number, length, and distance between two ramifications varies. Along with other morphological criteria, these components determine the compactness of the bunch [11]. The stem accounts for 3 to 7% of the total bunch weight, depending on the grape variety, number of grapes on the bunch, and its sanitary state [1,2,12].

### 2.2. General Composition

**Figure 2:**
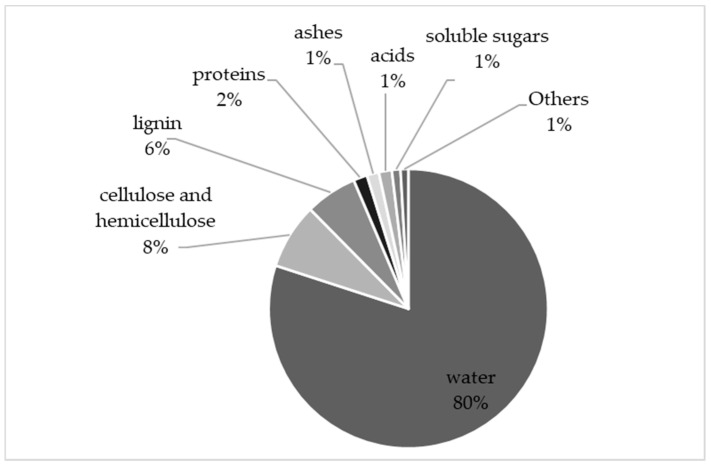
General composition of grape stems.

This part of the review aims to summarize the main compounds found in grape stems. An estimation of their quantification based on the available data is shown in Figure 2. This composition is close to the one described by Foulonneau et al. which is similar to that of the vine’s leaves and tendrils [2]. For each type of compound, available data were summarized. It should be noticed that the comparison of published data is difficult, as their proportions can be impacted by different factors, such as grape variety, vintage, maturation state, as well as differences in extraction techniques and units.

#### 2.2.1. Water

As the stem’s main component, water accounts for 55 to 80% of stem weight [2,3,13,14]. In 1976, Rice et al. measured the moisture of fresh grape stems from ten grape varieties, five reds (Concord, Ives, Baco noir, Red hybrids, and Cascade) and five whites (Aurore, Concord CP, Delaware, Niagara, and Catawba) planted in New York state, USA [14]. The values ranged between 68.4 and 79.1% of stem fresh weight (FW). No significant differences were found between red and white grapes and variability was imputed to the grape variety. In 2010, Gonzalez-Centeno et al. studied the overall stem composition of ten other grape varieties, six reds (Cabernet Sauvignon, Callet, Manto Negro, Merlot, Syrah, and Tempranillo) and four whites (Chardonnay, Macabeu, Parellada, and Premsal Blanc) planted on Mallorca Island, Spain, and found similar values, ranging from 55 to 80% of FW [13]. Of the grape varieties studied, white grape varieties appeared to have significantly higher moisture content (71.7 g/100 g FW) than red varieties (62.5 g/100 g FW). Stem water content appeared to depend on the grape variety. However, none of these studies considered stem maturity, which could have a major influence on the values.

#### 2.2.2. Cellulose and Hemicellulose

In stems, as in classical vegetable biomass [15], cellulose is the most abundant biopolymer followed by hemicelluloses (mannans, xyloglucans, xylans) [13,16,17,18,19]. Cellulose content values range from 12 to 38% dry matter (DM) (Table 1). The observed large variability might relate to differences in analytical procedures (extraction, analyses, and calculation) [17] and/or variability between grape varieties [20].

#### 2.2.3. Lignin

Lignin content ranges from 13 to 47% of DM (Table 1), with many studies reporting on the variability and providing different explanations, such as analytical method [17,18], grape variety [16,18], or stem maturity [21]. Indeed, studies have used different measurement and calculation methods to evaluate the lignin content, with some including acid soluble and insoluble lignin [16,17] and others including only acid-insoluble residues as the amount of lignin [18]. These method variations can lead either to an over- or under-estimation of total lignin content. The stem’s ripening speed depends mainly on the grape variety and climatic conditions [22]. Full lignification often occurs beyond berry maturity [1]. Indeed, the maturity stage of the stem at harvest will affect its composition. To our knowledge, stem maturity has not been considered in previous studies. It would be interesting to evaluate this maturity to better understand stem composition evolution during maturation.

#### 2.2.4. Proteins

Stem protein content ranges from 5 to 11% DM (2–3% of fresh weight) with a mean of 7% [13,18,21,23] (Table 1). The obtained values do not consider whether variations are related to grape variety or only to the biological variability of the raw material induced either by stem maturity or the extraction process (drying, crushing, etc.). These values are consistent because stems are not vine storage organs. Notably, different studies mention the presence of resistant proteins, referring to proteins bound with lignin, which are difficult to access, suggesting that the protein level could be underestimated [21,23]. Total protein quantification is, therefore, complex.

#### 2.2.5. Ashes

As with protein content, reported ash content is relatively consistent across different studies, with a mean value of 6.9% DM, regardless of the grape variety or origin (Table 1). Prozil et al. used inductive coupled plasma (ICP) to analyze detailed metal cation composition and identified potassium as the main mineral element of grape stems (K: 0.9%, Ca: 0.15%, Mg: 0.02%, Zn: 0.01% and Na < 0.01% of total ash content) [18].

#### 2.2.6. Acids

Stem acidic composition has been estimated by measuring stem extracts’ total acidity using a reaction with Bromothymol blue, with values ranging from 13.5 to 15.0 g/kg FW, or approximatively 1 to 2% of the total stem weight [2]. No information regarding further analysis of acid types was found in the literature.

#### 2.2.7. Sugars

Stems have a low sugar content [2,21]. According to Gonzalez-Centeno et al. soluble sugar content, determined as glucose, according to the Haas colorimetric method (which uses anthrone as the reactive and measures the absorbance at 620 nm), ranges between 1.8 and 3.7 g/100 g stem FW [13]. Sugar concentration variability is related to grape variety rather than color. Similar values were found in other studies: 1.70% for Manto Negro [23], 1.04% for Premsal Blanc [21], with soluble sugar content lower than 10 g/kg of stem FW [1]. Therefore, stems do not represent a significant sugar input for fermentation compared to grape berries (sugar content 14.9 g/100 g FW) [13].

The main components of grape stems and their respective concentration, as described in the literature, are summarized in Table 1.

### 2.3. Polyphenolic Composition

Phenolic compounds are widely present in the plant kingdom, and red grape varieties contain a high concentration of these compounds, especially in grape solid parts, skin, and seeds. Studies have also reported their presence in vine-shoots [25,26,27]. Stem extract analysis found that stems are rich in polyphenolic compounds [28,29], with intermediate concentrations between the higher concentrations in grape seeds and the lower concentrations in grape skins [30].

#### 2.3.1. Total Phenolic Content

The Folin–Ciocalteu method is a common technique for estimating total polyphenolic content in a vegetal fraction. Gallic acid is used as the standard and results are reported in gallic acid equivalent (GAE). Table 2 presents data from the literature on white and red grape varieties. To allow for comparison between the reported results, the unit was standardized (mg GAE/100 g DM). Grape stems show a wide range of total polyphenolic content. In white grape varieties, total polyphenolic values range from 400 to 22,900 mg GAE/100 g DM and in red grape varieties, similar values were found, ranging between 348 to 38,400 mg GAE/100 g DM. Variability in total polyphenol content can be attributed to many factors. Grape variety is one of these factors, as shows the important difference between the content measured in Asyrtiko (1248 and 1115 mg) and Athiri (400 and 480 mg) grape varieties by Anastasiadi et al. in two consecutive years [29], or in Chardonnay (4764 mg), and Pemsal blanc (9002 mg) by González-Centeno et al. [31]. Furthermore, Anastasiadi et al. and several other studies have also observed an important effect of vintage [29,32,33]. The importance of vineyard localization on the polyphenolic content was highlighted by the study of Spatafora et al. and Gouvinhas et al. demonstrated the effect of altitude [33,34].

In addition to the effect related to the grape and its growing conditions, several authors highlighted the impact of the extraction method on total polyphenolic content in the same grape variety. Makris et al. obtained values ranging from 3120 to 7468 mg GAE/100 g DM for Roditis grape variety, merely by changing the composition of the extraction solution [35,36,37]. Jimenez-Moreno et al. obtained similar results on Mazuelo stems, with values ranging from 1276 to 51,045 mg GAE/L/100 g of DM. They highlighted the fact that ethanol concentration was the most determinant parameter among temperature, solid/solvent ratio and ethanol content [38]. Interactions between these three different extraction parameters were also found. The size of the stem particles used for extraction also had an impact on the total polyphenol content, where smaller particles increased the exchange surface, allowing better extraction [39].

Further investigation to develop a standardized extraction method could help to compare results, including factors linked to either the grapes (e.g., grape variety [31,32,37,40,41], vintage conditions [29], vineyard localization [31,42], stem maturity [43]) or to differing extraction parameters [36,37] (e.g., solvent, duration, stem particle size, temperature).

#### 2.3.2. Non-Flavonoid Compounds

The total phenolic content comprises an important variety of molecules containing phenol rings in their chemical structure. Polyphenolic compounds are divided into two major categories, flavonoids and non-flavonoids. The presence of both types of molecules has been studied in grape stem extracts, with detailed molecular compositions reported in the literature.

**Figure 3:**
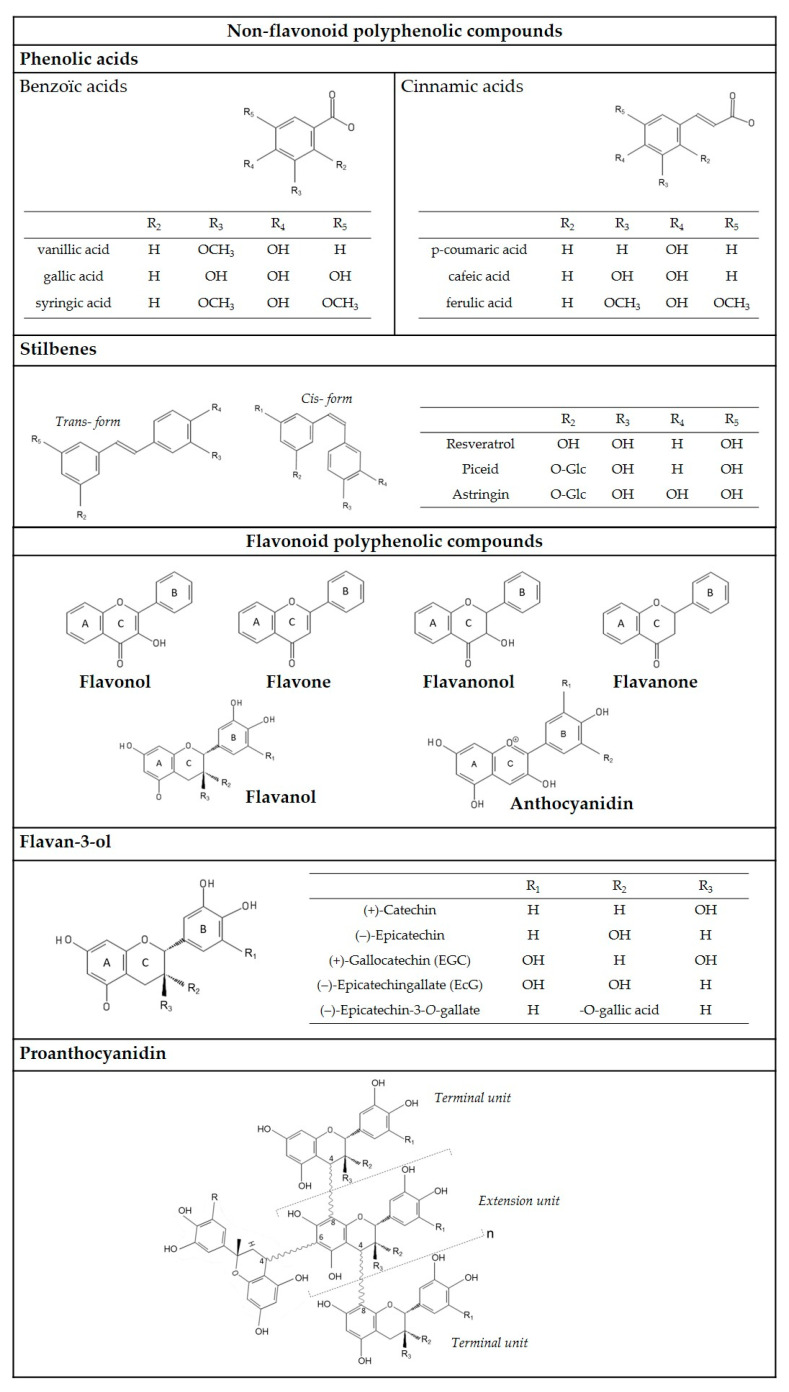
Non-flavonoid and flavonoid polyphenolic compound of grapes and wine.

Non-flavonoid molecules include phenolic acids, stilbenes, and hydrolysable tannins (Figure 3). To our knowledge, no hydrolysable tannin content has been reported in the literature.

##### Phenolic Acids

Different phenolic acids have been identified in grape stem extracts: caftaric acid [28,36,37,39,40,44,47,48], coutaric acid [28,40], gallic acid [19,28,29,34,38,39,48,49], coumaric acid [19,28,29,41], caffeic acid [29,41], syringic acid [19,28,29], ferulic acid [29], protocatechuic acid [48], *trans*-cinnamic acid [48], and other unidentified hydrocynnamic acids [44,47]. Many of these acids have also been identified in other grape parts, such as pulp, skin or seeds, or in other wine by-products, such as pomace [28,40,50].

Some authors have quantified these different phenolic acids using HPLC techniques. Results for white and red grape varieties are shown in Table 3. When the units differed, the values were standardized by converting to mg/kg DM. Comparing these values across studies remains difficult because extraction protocols differed according to the study and not all papers quantified phenolic acids. However, the major phenolic acids in stems appear to be caftaric acid and gallic acid. Caftaric acid was found in a concentration between 5.1–12,820 mg/kg DM in white grape varieties and 12.5–1500 mg/kg DM in red varieties. Gallic acid reached concentrations of 30–469 mg/kg DM in white varieties and 6.5–300 mg/kg DM in red varieties. As for the total phenolic content, individual phenolic acid concentrations are influenced by the grape variety [28,29,44], geographical origin [34], and the vintage [29]. The study of Gouvinhas et al. shows that the concentration of phenolic acids, such as caftaric and hydroxycinnamic acids, are highly correlated to the altitude and the vintage [33]. A strong thermal and water stress, related to the lower altitude, increases the synthesis of phenolic compounds in the plant and consequently in the stem. The effect of water stress is well demonstrated in the study of Alonso et al. where, in the no irrigated variants of Tempranillo and Syrah stems, the concentration of major phenolic acids, namely caftaric and coutaric acids, are significantly higher than in the irrigated variants [28]. In wine, these compounds have no odor, nor aroma. However, they can be precursors to volatile phenols, molecules that can induce defects in wines. Their extraction from stems is not necessarily interesting for winemaking.

##### Stilbenes

*Trans*-resveratrol and ε-viniferin are the two main stilbenes identified in grape stem extracts [29,33,34,36,38,41,44,47,48,49,51]. Values found in the literature are summarized in Table 4. *Trans*-resveratrol values ranged from 31 to 393 mg/kg DM and ε-viniferin, value range from 1.91 to 900 mg/kg DM. According to several authors, the differences between the values are mainly due to the different cultivars, geographical regions, and vintages [29,33,52]. As mentioned in Piñiero et al.’s study, the extraction protocol has a great impact on the extraction yields, especially the ethanol concentration and the sample-solvent ratio [52]. On the other hand, extraction duration (between 15 and 35 min) did not have a significant impact. Bavaresco et al. studied the transfer of stilbenoid compounds in wine in extraction conditions similar to wine (11% (*v*/*v*) ethanol and 250 ppm (*v*/*v*) methanol) and found only *trans*-resveratrol in the extract. The values ranged from 6.0 to 17.8 mg/kg DM, meaning that the level of stilbenes potentially extractable during maceration would be lower than the values found in the extracts presented in Table 4. The presence of other stilbenoid compounds, such as piceatannol, was also mentioned in the literature [41,52]. Finally, some studies reported that no stilbenes were found in the stem extracts [37,44]. This could either be related to the extraction method or to grape stem composition variability.

#### 2.3.3. Flavonoid Compounds

Flavonoid compounds share the same basic structure formed by two aromatic rings linked by three carbons: C6-C3-C6. This group of molecules includes flavonols, flavanols, flavanonols, flavones, flavanones (intense yellow pigments), and anthocyanins (red or blue pigments). Flavan-3-ols form oligomers and polymers, called proanthocyanidins or condensed tannins. Their different structures are presented in Figure 3. The most common flavonoid compounds in grapes and wines are flavonols, flavanols, anthocyanidins, and their derivatives.

Only one recent study tentatively identified a flavone in stem extracts, chrysoeriol malonyl-apiosyl-glucoside [36]. To our knowledge, no other flavones or flavanones content has been reported in the literature.

##### Flavonols

The different flavonols identified in grape stem extracts are quercetin 3-O-glucuronide [34,36,37,40,44,47,49], quercetin 3-O-glucoside [29,34,40,41], kaempferol 3-O-glucoside [36,40,44,47], myricetin 3-O-glucoside [40], myricetin 3-O-glucuronide [40], quercetin 3-O-rutinoside [37,39,44,47,48,49], quercetin 3-O-galactoside [29], quercetin 3-O-rhamnoside [29], kaempferol [29], quercetin [29], isorhamnetin-3-O-(6-O-feruloyl)-glucoside [44,47], and kaempferol-3-O-rutinoside [44,47].

Different authors have reported concentration values of these compounds for various white and red varieties (Table 5). Quercetin derivatives were reported to be the main flavonols followed by kaempferol derivatives. Quercetin-3-O-glucuronide, quercetin-3-O-rutinoside and quercetin-3-O-galactoside appeared to be the most abundant flavonols in grape stem extracts, depending on the extraction solvent used for sample preparation. The solubility in water of flavonol derivatives increases in the following order: rhamnoside < glucoside < galactoside < glucuronide < rutinoside [54]. Using only water for the extraction Kosinska–Cagnazzo et al. found only quercetin-3-O-rutinoside in the extracts, and the quantity varies with the size of the stems [39] when Barros et al. and Leal et al. with 50 and 70% of methanol in water extracted meanly quercetin-3-O-glucuronide [44,47]. The addition of organic solvent allows for the extraction of more apolares substances, such as kaempferol derivatives. However the high amount of organic compounds in the extraction mixture, as the 90% acetonitrile used by Anastasiadi et al. could conduct to the loss of the water-soluble derivatives [29]. This demonstrates the importance of the extraction conditions on the profile and the quantity of polyphenols measured in stems.

##### Flavanonols

Astilbin [36,37,40,49] and engeletin [40] are the two main flavanonols identified in grape stem extracts. Only Souquet et al. quantified astilbin (35 mg/kg of stems) and found traces of engeletin in the stem extracts [40]. Dihydroquercetin, also called taxifolin, is the flavanonol mainly identified in grapes and wine, and was not found in grape stem extracts.

##### Flavan-3-ols and Proanthocyanidins

The profile of flavan-3-ols and proanthocyanidins was measured in the stem extracts using HPLC-DAD or HPLC-MS techniques. Information about molecular ion and the typical fragments are summarized in Table 6.

Among flavanols monomers, many studies reported the presence of catechin and epicatechin in grape stem extracts. Epicatechin gallate was found in two studies [29,47]. To our knowledge, no epigallocatechin was identified in grape stem extracts as a monomer unit.

Proanthocyanidin dimers and trimers were identified in stem extracts using the HPLC-MS technique: dimers B1, B2, B3, B4, B1-3-O-gallate, B2-3-O-gallate, B3-3-O-gallate, and trimers T2 and C1.

The three main compounds found in the stem extracts are catechin and the dimers B1 and B3 (Table 7). The proportion of all compounds seems to depend on the grape variety and the vintage, but also on the study. The choice of extraction conditions and analytical method essentially influences the profile reported by the different authors. This can be the reason why Alonso et al. did not find catechin in all extracts [28], when others found catechin content ranging from 50 to 7640 mg/kg DM. The epicatechin content is low compared to catechin content. Barros et al. reported the sum of catechin and epicatechin in the extracts, with values ranging from 22 to 32 mg/g DM, depending on the grape variety [47]. In general, the concentrations of dimers B1 and B3 were found in the same magnitude as catechin, from 133 to 1958 mg/kg DM, and from 41 to 993 mg/kg DM, respectively.

Procyanidin B1 has been reported as the main oligomer in skins [58,59,60], whereas procyanidin B2 [60,61,62] is the main oligomer in seeds. Therefore, the phenolic composition of stem extracts is likely to be closer to grape skins.

Proanthocyanidins or condensed tannins are present in plants in different degrees of polymerization. When this degree is higher than three, these compounds cannot be quantified by actual HPLC-MS methods. Total proanthocyanidin content can be estimated by several methods. The Bate–Smith reaction is most commonly used and is based on the ability of condensed tannins to be depolymerized under acidic conditions. This chemical depolymerization, followed by auto-oxidation, generates anthocyanidins, hence they are also called “proanthocyanidins” [63]. The concentration of the resulting colored molecules can be measured by spectrophotometry to estimate the quantity of monomers included in the condensed tannins. Other techniques use a reaction between the nucleophile site of the tannin and an aldehyde, such as vanillin or DMACA, to produce a colored product where the measured intensity increases with the quantity of tannins, but decreases with the polymerization degree of the tannins, as only the terminal monomer is reactive. The DMACA method is based on the reaction between catechin and 4-dimethylaminocinnamaldehyde, resulting in the formation of a blue complex that absorbs red light (around 640nm). In the vanillin assay, vanillin is protonated in an acidic solution and reacts specifically with the flavan-3-ols, dihydrochalcones, and proanthocyanidins, producing a red-colored compound where the concentration is measured by spectrophotometry at a wavelength between 500 and 550 nm. In this case, catechin is often used as a standard. Methylcellulose precipitation method allows proanthocyanidic polymers to be selectively precipitated with methylcellulose (MC), with which they form insoluble complexes. The MC plays the same role here as the salivary proteins in tasting. Ammonium sulfate (NH_4_)_2_SO_4_ in the reaction medium increases its polarity, thus promoting complex insolubilization and precipitation. For the protein precipitation method, a known amount of protein (BSA) binds to the tannin in the sample, forming a protein–tannin complex that precipitates. Then, precipitate is washed by a ferric chloride solution, which forms a colored complex, the absorbance of which can be read on a spectrophotometer at 510 nm. The amount of color is proportional to the amount of tannins in the stem extract [64].

Values obtained for different grape varieties are presented in Table 8, classified according to the analysis method. As expected, different methods produced sensibly different results. Values are variable, even using the same analysis method, and these differences could not be linked to the grape color. Moreover, for the same grape variety (Premsal blanc), values found in two different studies were significantly different: 79.0 mg/g DM in Llobera et al. and 181.4 mg/g DM in Gonzalez-Centeno et al. [21,31]. Makris et al. showed that the extraction method can modify the measured total proanthocyanidin value for the same grape variety by a factor of 5 [35].

Based on the values obtained by the Bate–Smith method (expressed in mg/g DM), proanthocyanidins appear to be the most abundant type of polyphenols in stem extracts. The dimer concentrations shown in Table 7 (expressed in mg/kg DM) represent only a small proportion of the proanthocyanidin content. The high values of total proanthocyanidin content suggest an abundance of polymerised forms, which is confirmed by the results of the mean degree of polymerization (mDP), which was found to be higher than 4.6 for all studied grape varieties (Table 9).

Thiolysis or phloroglucinolysis are used to analyze the condensed tannin composition. These reactions are depolymerization methods that cut the polymers into subunits. Only the extension unit forms adducts with the reactive, allowing for differentiating them from terminal units. The different monomers can be separately quantified by HPLC and the mean degree of polymerization can be determined. Results reported in the literature are presented in Table 9. The experimental values of the mean degree of polymerization (mDP) range from 4.6 to 10.2. The general composition shows that epicatechin is the main unit of the polymerized proanthocyanidins; it is also mainly found in extension units, whereas catechin is mostly found in terminal units. Merlot and Chardonnay were studied in two different papers, with different analysis methods, and the mDP were slightly different; higher for Souquet et al. than for Gonzalez-Centeno et al. [31,40]. This difference could be explained by vintage conditions, vine location, and analytical techniques. Sensitive difference can be observed between the two methods; EcG and EgC were found in higher concentrations using the thiolysis method than using phloroglucinolysis.

##### Anthocyanins

Anthocyanins are mainly located in grape skins [2,30]. However, recent studies analyzing different grape varieties identified some anthocyanin compounds in grape stem extracts: malvidin-3-O-glucoside [47,48], malvidin-3-O-(6-O-caffeoyl)-glucoside [47], malvidin-3-O-galactoside [48], and malvidin-3-O-rutinoside [47]. Total anthocyanin content of the stem extracts ranged from 0.06 to 1.4 mg/g of DM, and these compounds were not detected in some varieties. The concentration in anthocyanins was low compared to other flavonoid contents.

#### 2.3.4. Impact of Polyphenolic Composition

Polyphenolic compounds have been widely studied and, apart from their influence on wine color and structure, they can influence different parameters, such as astringency or antioxidant activity.

##### Astringency

Astringency produces a contraction of the buccal mucosa when salivary proteins form complexes with tannins. Salivary amylase reacts strongly with astringent compounds and causes the mouth dryness sensation.

The influence of grape stem extracts’ polyphenolic composition on astringency has been studied using ovalbumin as a precipitation agent and tannic acid as a standard. Ovalbumin mimics the salivary proteins and quantifies astringency related to the precipitation of polyphenolic compounds and saliva. The results showed that stem extract astringency increases with maceration time and remains stable after 4–5 days [43]. Three maturation stages were studied, and ripening appeared to increase proanthocyanidin extraction during maceration and decrease the astringency of the extracts of all cluster parts. One hypothesis for the decreased astringency was a decrease in the mDP of proanthocyanidin extracted during stem ripening [43]. The relation between quantity, mDP, percent galloylation, and percent trihydroxylated units of proanthocyanidin and astringency has been studied in many wines and seed extracts [43,50,65,66]. In red wines, weaker astringency was found for lower mDP. In addition, the mDP is an average of the degree of polymerization and does not give clear indications of the proportion of polymeric and oligomeric proanthocyanidin content. Li et al. showed that polymeric polyphenols react more strongly with salivary proteins than oligomeric ones, inducing a higher sensation of astringency. During the ripening of the stems, the proportion of oligomeric forms may increase and could explain the decrease in astringency. The variation in proanthocyanidins, according to grape variety and stem ripening stage, appears to have a great influence on sensorial perception, especially regarding astringency. It would be interesting to study this parameter further when stems are kept during winemaking.

##### Antioxidant Activity

The antioxidant potential of polyphenolic compounds can be measured by different methods [67]. Studies have used different measurement techniques to characterize the antioxidant potential of stem extracts, such as 2,2-azinobis(3-ethylbenzthiazoline-6-sulphonic acid) (ABTS), 1,1-diphenyl-2-picrylhydrazine (DPPH), ferric reducing antioxidant power (FRAP), cupric reducing antioxidant capacity (CUPRAC) [31], oxygen radical absorbance capacity (ORAC) [31], and superoxide radical scavenging activity (O_2_^·-^) [47]. ABTS, FRAP, and DPPH appeared to be the most used methods. Antioxidant capacity measured by DPPH can be expressed in different ways: either as the quantity of antioxidant necessary to decrease the concentration of initial DPPH or using a reference such as trolox. Summarizing the available data and comparing the values is not straightforward. Therefore, only the ABTS and FRAP values found in the literature are presented in Table 10.

As mentioned by Gonzalez-Centeno et al. it is difficult to compare the values reported in the literature because there is no standardized method to characterize the antioxidant potential; extracts are obtained using different techniques and the results are expressed in different units [31]. According to the literature, ABTS and FRAP results usually show good positive correlation [31,68,69,70,71,72]. Despite the difficulties of cross-study comparisons, all studies reported that stems can be a good source of antioxidant compounds.

### 2.4. Aromatic Composition

The use of stems during winemaking has been reported to bring vegetal and green aromas to the wine. Different studies focused on the aromatic compound found in stem extracts. In 1997, Hashizume et al. listed eight different green odorant compounds detected in grape stems from Cabernet Sauvignon and Chardonnay grape varieties: hexanal, (E)-2- hexanal, (Z)-1,5-octandien-3-one, 2-methoxy-3-isopropylpyrazine, 2-methoxy-3-isobutylpyrazine, dodecanal, (E,Z)-2,6-nonadienal, and an unknown compound (Table 11) [73]. These compounds were the same in both grape varieties. For Cabernet Sauvignon, four other aromas (a cooked vegetable-like odorant, a burned bamboo-like odorant, a sweaty unpleasant odorant, and a floral aroma) were also found during the extract analysis but were not analyzed because the study focused on vegetal aromas. Quantifying each compound showed that (Z)-1,5-octandien-3-one was the main green odorant compound from stems. These extracts were also compared to leaf, berry, and skin + seed extracts and stems appeared to contain the highest proportion of methoxypyrazine. Roujou de Boubée et al. focused on 2-methoxy-3-isobutylpyrazine (IBMP) in the Cabernet Sauvignon grape variety to determine the localization of this aromatic compound within the grape cluster, and found that stems were richer in IBMP, confirming the results of the Hashizume research team [73,74]. They also studied IBMP location during ripening and showed that it decreases in stems and seeds but increases in skins. Matarese et al. studied the entire fraction of volatile compounds of ground stems and other grape plant parts and reported that geraniol and geranic acid were the two main monoterpenes of the stem volatile fractions [75]. Other compounds, such as linalool and nerol, were also identified but in smaller proportions.

Ruiz-Moreno et al. performed a GC-olfactometry and a GC-MS on Syrah stem extracts and found more than 80 odorant zones (OZ) [76]. Among them, eight OZ were found to be predominant and GC-MS identified the responsible molecules (Table 11). This study specified that stem extracts have a similar composition to that of wine in terms of aromatic compounds and should have a quantitative rather than qualitative effect if added to the wine.

In 2016, a study of Cabernet Sauvignon stems found a large amount of 1,8-cineole in the stems compared to the grape berries, and that this quantity substantially decreased during ripening [77]. Larger amounts of 2-methoxy-3-isopropylpyrazine (IPMP) and IBMP were found in the stems than in the grape berries. Again, these amounts decreased with ripening. Finally, this study identified methyl salicylate, which is reported to have a fresh and minty aroma and has higher levels in stems than berries (250 times higher).

Stems appear to be a rich source of valuable compounds, including polyphenols. Considering waste in wine production, their availability is high at harvest time and, as reported in the literature, grape stems may provide a cheap source of these compounds of interest. Recent studies have examined stem extracts for human health applications [44,48,78]. Although the destemming technique is widespread, some winemakers keep the stems during the winemaking process.

## 3. The Use of Stems during Winemaking

As explained in the introduction, in most cases of white winemaking, stems are kept for pressing and removed with the pomace. Because stems act as drains, keeping them during pressing induces better juice extraction. According to the literature, it also limits the presence of the thermo-unstable proteins responsible for protein breakdown [1]. For red winemaking, the impact of keeping the stems during fermentation and maceration appears to be more empirical. Understanding which elements are transferred and what impact they have on the wine is essential for advising winegrowers on this practice.

When stems are included, the whole cluster addition is the most common technique used. However, to precisely study the impact of stems, researchers have often added the stems back in the tank after destemming [3,12,30,43,57,73,79,80,81,82,83,84]. Different proportions of stems and whole clusters [81,83,84,85], different maceration time [30,43,79], and different stem pretreatments [3] have been used. Some studies also tested the use of stem powder [39] or stem extracts [76,86] as an oenological additive compound.

### 3.1. Impact on the Winemaking Process

Adding stems or using whole clusters can increase the must volume by 30%, which has a technological impact on the vatting and the maceration phases. In addition, a higher pressuring capacity is required [1].

To our knowledge, the impact of stems on alcoholic and malolactic fermentations (MLF) is barely described in the literature. Comparing destemmed and full-clustered musts has shown that must containing stems start fermenting faster, resulting in a wine with fewer residual sugars [1]. This may result from the structural configuration of stems, which allows higher incorporation of oxygen in the must, encouraging yeast proliferation. Moreover, the presence of such structures acting as temperature buffers could reduce temperature variations and hence prevent stuck fermentations. These effects are different depending on the volume of whole clusters or stems added. A recent study showed that 20% of whole clusters or 3% of stem weight in the vat did not influence either the temperature or the alcoholic fermentation kinetics [84]. For both fermentations, further studies are needed to better understand the impact of stems on microbial activity and kinetics.

### 3.2. Impact on the Main Wine Compounds

Stems release compounds, such as must [43,57,80,81], in a matrix, which then interact with the other grape-extracted compounds (from berries and seeds) and provoke a change in the overall balance of the final wine. Among the different types of compounds found in wines, some are widely influenced by the presence of stems, including ashes, acids, alcohols, and phenolic compounds.

#### 3.2.1. pH and Acid Composition

Summarized data on the pH and acidic composition of wine made with and without stem addition are presented in Table 12.

Hashizume et al. found an increase in pH for Pinot Noir and Muscat Bailey A musts when incorporating the stems back in the vats [3]. This phenomenon was also observed by Pascual et al. on Cabernet Sauvignon musts and more recently by Casassa et al. during Pinot Noir winemaking using either whole clusters, raw or dried stems [12,83,84].

However, pH increases are not always significant. These differences in terms of variations may be linked to the high buffering capacity of wine matrices over acido-basic balance that mainly depends on the grape variety [50]. The impact of stem contact duration has also been studied. For Castelao musts, Spranger et al. reported that pH did not show a significant increase after seven days, but showed a significant impact after 21 days of contact [79]. Therefore, contact duration seems to influence pH variation.

According to the acid composition, titrable acidity was shown to be significantly lower than the control samples for Cabernet Sauvignon [12] and Pinot Noir wines going through stem contact [84]. However, this finding seems to depend on how the stems are incorporated in the Casassa et al. latest study; the use of whole clusters did not show a significant decrease in titrable acidity [83].

Changes in acid composition appear to be responsible for these pH variations in wine. More specifically, tartaric acid, which is the most abundant acid found in wines and musts, seems to be affected by the addition of stems, but less by the use of whole clusters (Table 12). After seven days of contact, its concentration was lowered by 4% for Castelao wines [79], 9% for Muscat Bailey A [3] and 10% for Pinot Noir wines [3]. With longer stem contact duration, the decrease in tartaric acid was greater (from 4% after seven days to 7% after 21 days for Castelao wines [79]). This loss could result from precipitation mechanisms of tartaric acid. As noted earlier, stems are rich in mineral compounds, especially potassium, so their interaction with tartaric acid is a possible explanation [12].

Other acid concentrations might also be affected by stem contact as a result of molecular interactions. For instance, lower concentrations of succinic acid were reported for Muscat Bailey A (−16%), whereas phosphoric acid concentration was increased by 38% [3]. Differences in lactic and malic acid concentrations mainly resulted from the winemaking process and whether the MLF was performed. Lastly, Casassa et al. highlighted the fact that the use of the whole cluster may increase the volatile acidity of the wine potentially due to undesirable bacterial growth taking place in the air spaces within the whole clusters [83].

#### 3.2.2. Ashes

Only a few authors have studied the impact of stems on the mineral composition of wines. However, both Hashizume et al. and Sun et al. found significant increases in the concentration of several mineral ions [3,80]. The related values are summarized in Table 13.

Both authors found that stem addition increases potassium (K), phosphorus (P), and calcium (Ca) concentrations. This reinforces the hypothesis of tartaric acid precipitation inducing a pH increase.

Some variations were recorded depending on the grape varieties, especially for magnesium (Mg), sodium (Na), copper (Cu), and zinc (Zn), indicating that the impact of stems could also depend on the grape variety.

#### 3.2.3. Ethanol Content

Several authors related the addition of stems to a lower wine ethanol content [12,80,84] (Table 14). Hashizume et al. attributed this decrease to a dilution phenomenon [3]. Indeed, stems have a high water content (see Section 2.2.1. Water), which could be transferred to the wine during maceration. Pascual et al. presented similar conclusions and added that the stem surface could also capture ethanol molecules by adsorption [12].

Nevertheless, lower ethanol values are not always clearly observed. While comparing the impact of different technologies on the wine profile, Spranger et al. did not observe any real change when adding stems to the fermenting wines [79]. The impact of stems on alcohol content is not yet well understood; it might be interesting to compare these results with the moisture content of stems.

### 3.3. Impact on Polyphenolic Composition

#### 3.3.1. Total Phenolic Compounds

Two methods were used to analyze the total phenolic fraction of wines: Folin Index (FI) and Total Polyphenol Index (TPI). The FI is based on the Folin–Ciocalteu method. TPI uses the typical properties of the benzenic structures found in phenolic compounds, which can absorb at 280 nm when measured by spectrometry. Even though this measure is not very accurate for quantification, it gives a good indication of the phenolic content in wines. Several types of phenolic compounds contribute to this index, such as anthocyanins and tannins, as well as a small fraction of non-phenolic compounds [50]. Data available in the literature are summarized in Table 15. For most of the grape varieties, the total phenolic content increased when stems were included during winemaking. Castelao is the only grape variety for which no significant difference was found. The magnitude of the variation seems to be correlated both to varietal differences and maceration duration.

#### 3.3.2. Non-Flavonoid Compounds

Even if some interesting non-flavonoid compounds were found in the grape stem extracts, their transfer and presence in wines has not been thoroughly investigated. Pascual et al. examined hydroxycinammic acid derivatives in Cabernet sauvignon wines, and Benitez et al. in Palomino fino [12,85]. These compounds were measured using reversed-phase HPLC, diode array detector, electrospray ionization, and tandem mass spectrometry systems (HPLC-DAD-ESI-MS). Overall, the tested wines were not significantly affected by stem contact in terms of phenolic acid content. Caftaric and gallic acids that were the main phenolic acids found in grape stem extracts does not seem to be significantly transferred to wine (Table 16).

Although the concentration of stilbene and stilbenoid compounds has been extensively studied for the antioxidant properties in the stem extracts, to our knowledge, their transfer from the stem to the wine has not been studied. However, in their study, Bavaresco et al. mimicked alcoholic fermentation, using an hydroalcoholic solution (11% (*v*/*v*) ethanol and 250 ppm (*v*/*v*) methanol) as an extraction solvent, in order to quantify the content of potentially extractable stilbenes [51]. Their results showed that only *trans*-resveratrol was extracted. For this experiment, the ethanol content remained constant during the extraction. It would be interesting to carry out the same study with an increasing concentration of ethanol and also in fermenting must in order to valid the transfer of these compounds to the wine.

#### 3.3.3. Flavonoid Compounds

To our knowledge, the impact of stem contact on flavones or flavanones content has not been reported in the literature. Pascual et al. [12] studied the impact of stem contact on the flavonol content of Cabernet Sauvignon wine. Their results showed a significant decrease in total flavonols, mainly resulting from aglycones, and they suggested that stems might absorb these compounds. Further study is needed to confirm this hypothesis. Apart from this study, no further information was found in the literature.

##### Flavan-3-ols and Proanthocyanidins

Total proanthocyanidin content results are shown in Table 17 and individual flavan-3-ol and proanthocyanidin composition results are shown in Table 18. The total proanthocyanidin content seems to significantly increase when either stems or whole clusters are kept during maceration, regardless of the grape variety. According to Casassa et al.’s latest study on Pinot Noir wines, the increase seems to be more or less correlated to the amount of stems in the vat, whether fresh or dry [83]. This observation is also valid for Suriano et al.’s results on Primitivo wines [81].

According to the flavan-3-ol monomeric and polymeric composition, the intensity of the variations differed across studies. It is hard to draw conclusions from these values because stem proportions added for alcoholic maceration and maceration duration varied. However, there was a tendency for higher concentrations of catechins, epicatechin, dimer B1 and B3 in nearly every study. For dimers B2 and B4, the concentration variation did not seem to depend on either the grape variety or the maceration duration.

Suriano et al. reported a clear increase in the concentration of catechin, epicatechin, epicatechin gallate, and procyanidins B1 and B3 that evolved to the quantity of full clusters present in the wines [81]. Conversely, gallocatechin and procyanidin T2 (not shown here) concentrations decreased when stems were added, which could be linked to adsorption by stem bodies or interactions with wine molecules.

Little information is available regarding the impact of stems on proanthocyanidin mDP [12,79,80]. Available results did not show significant difference induced by stem contact (data not shown).

##### Anthocyanins

According to several authors, stem contact during maceration decreased total anthocyanin concentration [12,30,79,80,81] (Table 19). Spectrophotometric measurements to determine anthocyanin concentration are possible thanks to chemical methods based on anthocyanin color properties. pH variation methods, such as the Puissant–Leon method are based on matrix acidification by HCl that cause a change in anthocyanin color [50,87]; the SO_2_-bleaching method is based on the discoloration of anthocyanins in the presence of sulfur dioxide [88]. These measurement techniques are only partially accurate, because they only quantify the sum of free anthocyanin and the part of the combined anthocyanins fraction that is sensitive to sulfur-dioxide bleaching [50]. However, among the wines tested with the same method, anthocyanin concentration decreases seemed proportional with the stem contact maceration duration, with Malbec wines studied by Ribéreau-Gayon and Mihlé the only exception; anthocyanin content decreased with stem addition but not proportionally to the contact duration [30]. When whole grape clusters were used, the concentration in anthocyanin was even lower and tended to decrease with an increasing proportion of full clusters [79,80,81]. Although molecular interactions between compounds from the stems and the musts could explain part of this anthocyanin loss, Ribéreau-Gayon and Mihlé rejected this hypothesis, because the addition of stem extract did not affect the anthocyanin concentration [30]. Instead, the authors explained this loss by the adsorption phenomenon provoked by the stem bodies on the anthocyanin molecules, similar to the explanation given by other authors [81]. However, this finding was not found in Casassa et al.’s latest study [83]. The anthocyanin content of wine made of 50% and 100% of whole cluster was not significantly different from the control wine (fully destemmed). The authors highlighted that the vintage conditions can have more of an impact on the anthocyanin content than the winemaking process; an additional argument shows that it would be relevant to evaluate and take into consideration the maturity of the stems.

Some authors studied the individual anthocyanin composition by HPLC analysis (Table 20). Anthocyanin 3-monoglucosides, which are the major anthocyanins in the tested wines, were the most affected by stem addition, where the malvidin-3-O-glucoside counted as more than 50% of the fraction. Studies led by Spranger et al. showed a 19.6% decrease in its concentration after seven days of stem contact, and this decrease was even more important (30.2%) with extended stem contact (21 days) [79]. Similar results were reported by Sun et al. and Suriano et al. who found decreases of about 17 to 18% [80,81]. A decrease in anthocyanin 3-monoglucosides was also reported by Pascual et al. but to a lesser extent (6.0%) [12]. Furthermore, the proportion of stems added had a smaller impact on the anthocyanin 3-monoglucoside concentration than the stem contact duration.

Other types of anthocyanins, combined with specific molecules, were affected by stems, including p-coumarylated anthocyanins that showed decreasing patterns depending on stem contact duration [12,81]. However, the quantity of stems in contact with the must did not significantly lower their concentration. Decreases in acetylated anthocyanins did not always reach significance.

### 3.4. Impact on the Wine Sensorial Characteristics and Aging Potential

#### 3.4.1. Color

Wine color measurements were mainly performed using color intensity calculation, summation of the absorbance at 420, 520, and 620 nm, and the hue ratio between absorbance at 420 and 520 nm. The results are shown in Table 21. Using stems during winemaking tended to decrease color intensity and increase hue, giving the wine a more reddish color, but this result was not always observed. In some cases, adding stems decreased the hue [30,81]. CIELAB measurements were also performed in some studies but results were not significantly different when stems were included during winemaking [79,80] (data not shown). Different explanations were offered for the color changes: dilution linked to stem water released in the must, pH modification allowing the transformation of anthocyanins into uncolored compounds, and possible adsorption of anthocyanin content by the stems [3,12,30]. The impact of stems on color intensity was more important for short maceration and tended to have no significant impact on long duration maceration [30].

Other work reported increased color intensity despite lower anthocyanin content [81]; this was explained as stems bringing more oxygen to the must and promoting condensation between anthocyanins–tannins–acetaldehyde, which is important for color stability.

#### 3.4.2. Aroma and Volatile Compounds

Spranger et al. and Benitez et al. studied the impact of various proportions of stems on the volatile fraction of wines [79,85]. With Castelao wines, 1-hexanol (grass odor) and ethyl decanoate (waxy odor) were the two compounds affected after 21 days of maceration with stems. Among all the other compounds identified in both studies, no significant effect of stem quantity was found on the concentration of molecules.

Among the 14 volatile compounds identified by Casassa et al. the concentration of β-damascenone, a nor-isoprenoid described as a fruity aroma enhancer, was higher when whole clusters where used during winemaking [84]. However, sensory analysis did not confirm these results because wines were described as less fruity. No green taste was identified in the wines, although compounds that give a green taste, such as 1-hexanol, isobutanol, or hexanoic acid, differed in concentration when stems were added. In Casassa et al. latest study, wines made of 100% whole clusters of grapes showed higher levels of ethyl cinnamate and benzaldehyde (spice and almond-like odor) and those in which dried stems were added exhibited higher levels of esters (potential fruity and floral odors) [83]. Sensory analysis confirmed these differences: 100% whole cluster wine had higher vegetal, cooked fruit flavors and spicy notes and wines made with dried stems had more herbal and fruity odors. Compounds known to bring a vegetal note to wine, such as methoxypyrazines, were not examined in this study.

IBMP is known to be easily extracted during pressing and maceration [74]. Because its concentration is high in stems, stem contact processes could increase IBMP levels in must and wine. Consistent with this hypothesis, Hashizume and Samuta identified methoxypyrazine compounds in wines from Cabernet Sauvignon and Chardonnay varieties fermented with and without stems [73]. Compared to wines made from fully destemmed bunches, stem contact wines had significantly higher concentrations of 2-methoxy-3-isopropylpyrazine (IPMP), 2-methoxy-3-sec-butylpyrazine (SBMP), and IBMP. Similar patterns were found in a more recent study on Sauvignon Blanc wines [82]. The results are presented in Table 22.

More precisely, IBMP was the only compound detected without stem addition at concentrations greater than 1 ng/L, suggesting that IPMP and SBMP were introduced by the stem contact [73,82].

Although values were the same order of magnitude, differences in concentration reported in the literature could be linked to winemaking practices involving various stem quantities in contact with the must. Moreover, the number of wounds provoked during grape harvest, the lignification state of stems, and potential varietal effects could be responsible for these variations. Further work is needed to investigate the implication of each factor in methoxypyrazine concentration in stem-contact wines.

#### 3.4.3. Taste

Studies that performed sensory analysis of wines made using either stems or whole clusters reported similar conclusions regarding bitterness and astringency. Pascual et al. reported that the stem significantly increased astringency and tended to increase the bitterness of Cabernet Sauvignon wines [12]. More recently, Casassa et al. reported similar results for Pinot Noir wines, for both fresh or dried stems [83,84]. In these two studies, the degree of stem lignification was not considered. It might be interesting to examine whether the sensory impact is different when the stems are lignified.

On the other hand, in the conclusions of their latest study, Casassa et al. wrote about the impact of stems on the “freshness” of the wines [83]. This effect is relatively well known from an empirical point of view. In this study, the wines were noted as fresher, although no chemical compound could explain it. As mentioned before, methyl salicylate has been identified to bring a fresh and minty aroma [77]. It would be interesting to dose this compound in wines made with stems in order to see if it could explain the increased “freshness”. It seems that the stems have a simultaneous action on several factors, so the result is an increase in complexity and freshness of the wines. More studies seem to be needed to understand the complex effect of stems on wine quality.

#### 3.4.4. Wine Aging Potential and Stability

The impact of stem use on Tinta Miuda wine aging was studied by Sun and Spranger, who found that anthocyanin content was significantly affected after two years of bottle aging [89]. The anthocyanin content at bottling was higher for wines made without stems. It then decreased and reached a level where no significant difference could be detected between the two winemaking techniques. Individual anthocyanins were affected differently, where decreased delphinidin-3-O-glucoside, cyanidin-3-O-glucoside, petunidin-3-O-glucoside, peonidin-3-O-glucoside, and palvidin-6′′-O-acetylglucoside concentrations were not significantly affected by the presence of stems. Conversely, when stems where used, palvidin-3-O-glucoside and palvidine-3-O-glucoside-pyruvic acid adducts decreased more, whereas decreases in palvidine-6′′-O-p-coumarylglucoside and peonidine-6′′-O-p-coumarylglucoside were less important. The flavan-3-ols and the proanthocyanidins suggested that procyanidin B2, B3, B4, and C1 decreases were significantly lower when stems were used. Total oligomeric and polymeric proanthocyanidin contents showed lower decreases when stems were used. These results suggest that using stems during winemaking allows for a similar stability of anthocyanins as the control wine, but provides better stability of flavan-3-ols and proanthocyanidins. After two years of aging, mDP was significantly lowered but no significant differences were found between the stem and non-stem contact wine; the percentage of galloylation was not affected by the winemaking process or aging time; the total polyphenolic content stayed stable, suggesting that polyphenolic compounds were converted to other phenolic forms. No significant differences were found in wine color intensity, but the color of stem contact wine appeared more yellowish orange than the one of the non-stem contact wine.

Suriano et al. reported similar results regarding the anthocyanins content in Primitivo wines [81]. Fully destemmed grape wines showed higher anthocyanin content after 12 months of aging. For 25 to 50% of non-destemmed grape wines, color intensity increased, suggesting condensation phenomena between anthocyanins and tannins. No difference was found in the color shade after 12 months of aging, suggesting that using the whole harvest with stems could improve the color stability of wines during aging.

In Casassa et al.’s study, the Pinot Noir wines showed similar aging behavior after three and 12 months, regardless of the winemaking technique (added stems or 20% whole cluster) [84]; the anthocyanin content decreased, polymeric pigment levels increased, and tannin contents were stable. Same results were obtained for wine obtained using 50%, 100% whole cluster or dried stem [83]. Few studies have examined the impact of using stems on the aging potential. Excluding the rearrangement of anthocyanins classically observed in red wines, few conclusions can be drawn from these works. Although the antioxidant activity of stem extracts has been widely studied, the parallel with winemaking has not yet been sufficiently examined. If adding stems represents a source of antioxidants, it could be a way to reduce SO_2_. Two articles on this topic were found in the literature [76,86]. Ruiz-Moreno et al. showed positive results using stem extracts as a SO_2_ alternative in model solution for both antioxidant and antimicrobial action. In the other study, performed on red wines, Esparza et al. highlighted that the use of stem extracts could be a promising strategy to reduce SO_2_ in wines, but it still needs some optimization. In addition, a recent study on the use of grape stem extracts for protein precipitation showed that, in a model wine solution, these extracts could represent a good agent to remove unstable proteins [39]. Among the different grape varieties tested, Chasselas stem extracts, rich in polyphenols, showed the best results. Although used almost exclusively in red wines, it would be interesting to investigate the influence of stems on the protein stability of white wines.

Table 23 summarizes the main effects of the use of stems or whole clusters on wines from an oenological point of view.

## 4. Conclusions

Analysis of the available research has allowed us to highlight the main compounds that compose stems. Although they do not seem to contain any new specific compounds, the transfer of certain molecules such as metal ions, phenolic compounds, or even some aromatic compounds, may induce changes in equilibrium, and thus could explain the increase in aromatic complexity in some cases. Stems’ high phenolic compound content could make them good candidates for antioxidants and stabilizers. Stem composition was mainly studied to evaluate their potential use as a source of compounds of interest, particularly phenolic compounds and stilbenes, for other sectors, such as pharmacy and health. Consequently, stem extracts are often obtained through extraction procedures that involve treating the stems upstream (freezing, grounding, etc.) using strong organic solvents to produce good yields. This does not represent the extraction phenomena that could take place during the winemaking process. It would be interesting to approach the extraction procedures in a similar way to alcoholic fermentation and maceration processes. This would identify which stem components have a real impact on the must and the wine matrices.

For winemaking trials with stems, the variability of grape varieties and limited knowledge regarding stem maturity makes it difficult to compare the different studies. However, several points emerged, such as decreased alcohol content, increased pH, and decreased anthocyanins content. Very few studies investigated the impact of stems on the stability and aging potential of wines. It would be interesting to look further into the phenolic compounds present in the stem extracts and their antioxidant capacity.

In using whole bunches of grapes or only stems, it is important to consider the general state of the stems. Very few studies focused on stem maturity or the degree of lignification, which varies according to the grape variety, the terroirs, and the vintage. Using stems is not systematic for a winegrower; it depends on the conditions of the vintage. Therefore, it is appropriate to consider the state of the stems to acquire new knowledge and facilitate a better understanding of this winemaking technique.

## Figures and Tables

**Table 1:** Main chemical components of stems (values expressed in % DM).

Grape Variety	Cellulose	Hemicellulose	Lignin	Proteins	Ash
Pinot Noir [17] (Bellucci method)	24.65				7.66
Pinot Noir [17] (Sluiter et al. method)	25.3	13.95	47.29		7.66
Pinot Noir [17] (Goering-Van Soest method)	37.88	14.93	32.98		7.66
Red grapes [19]	34.6	14.5			
*Vitis vinifera* L. [18]	30.3	21	17.4	6.1	7.0
Cabernet Sauvignon [13]	23.0	11.6	12.8 to 22.6	5.8	10.8
Callet [13]	23.3	13.1		8.3	7.1
Manto Negro [13]	23.1	13.7		6.7	6.9
Merlot [13]	27.1	12.7		5.7	11.2
Syrah [13]	35.0	17.2		6.8	4.8
Tempranillo [13]	19.6	10.2		4.9	10.0
Chardonnay [13]	26.1	11.8		7.7	8.6
Macabeu [13]	25.0	13.6		6.6	5.5
Parellada [13]	26.3	14.0		11.2	6.4
Premsal Blanc [13]	22.2	9.8		9.2	5.9
Manto Negro [23]			31.6	7.29	5.48
Premsal Blanc [21]			22.91	5.12	6.94
Alsacian white grape variety [16]	36.3	24.5	39.6		3.9
Mix of Bonarda and Barbera [20]	12.19	25.7	32.35		6.11
Albariňo [24]	29.95	35.33	22.94		

**Table 2:** Total polyphenol content of stems of white and red grape varieties (mg GAE/100g DM).

Grape Variety	Vintage	Total Polyphenol Concentration
**White Grape Varieties**		
Aidani [29]	2009	1072.6
Aidani [29]	2010	722
Asyrtiko [29]	2009	1248
Asyrtiko [29]	2010	1114.6
Athiri [29]	2009	399.9
Athiri [29]	2010	480.8
Chardonnay [31]	2009	4764
Chasselas [39]	2015	300 to 4300 ^c^
Fernao Pires [44]	2017	11,015
French Colombard [32]	1987	2430 ^a^
French Colombard [32]	1988	1980 ^a^
Macabeu [31]	2009	7809
Malvasia Fina [44]	2017	12,309
Moscatel (Sanfins du Douro) [33]	2017	3235
Moscatel (Sanfins du Douro) [33]	2018	8305
Moscatel (Penajóia) [33]	2017	7802
Moscatel (Penajóia) [33]	2018	10,349
Moscatel (Medrões) [33]	2017	3793
Moscatel (Medrões) [33]	2018	8832
Moscatel [44]	2017	10,871
Parellada [31]	2009	8924
Premsal blanc [31]	2009	9002
Premsal blanc [21]	-	8730
Premsal blanc [45]	-	17,200 to 22,900 ^d^
Rabigato [44]	2017	9471
Roditis [35]	-	3120 to 7468 ^d^
Semillon [32]	1987	1950 ^a^
Semillon [32]	1988	1690 ^a^
Viosinho [44]	2017	9699
**Red Grape Varieties**		
Cabernet [39]	2015	1200 to 2000 ^c^
Cabernet Sauvignon [31]	2009	7076
Cabernet Sauvignon [28]	2000	2500 ^b^
Cabernet Sauvignon [34]	2009	348.0
Callet [31]	2009	11,525
Carnelian [32]	1987	2170 ^a^
Carnelian [32]	1988	1850 ^a^
Frappato [34]	2009	998.5
Mandilaria [29]	2009	1057
Mandilaria [29]	2010	1434.3
Manto Negro [31]	2009	8470
Manto Negro [23]	-	11,600
Manto Negro [45]	-	29,400 to 38,400 ^d^
Mavrotragano [29]	2009	1011.1
Mavrotragano [29]	2010	557.9
Mazuelo [38]	2016	1276 to 5104 ^b,d^
Merlot [31]	2009	4704
Merlot [39]	2015	900 to 2900 ^c^
Nerello Mascalese (Lingualossa) [34]	2009	2179.8
Nerello Mascalese (Milo) [34]	2009	4000.1
Nerello Mascalese (Santa Venerina) [34]	2009	1241.7
Nero d’Avola [34]	2009	2632.9
Ruby Cabernet [32]	1987	1950 ^a^
Ruby Cabernet [32]	1988	1730 ^a^
Sousao [46]	-	3135 ^a^
Syrah [31]	2009	9642
Syrah [28]	2000	2500 to 5000 ^b,e^
Syrah [39]	2015	200 to 2250 ^c^
Tempranillo [42]	-	4679
Tempranillo [31]	2009	7622
Tempranillo [28]	2000	1250 to 3750 ^b,e^
Voidomato [29]	2009	840.2
Voidomato [29]	2010	610

^a^ Unit mg GAE/100 g FM. ^b^ Unit mg GAE/L/100 g DM. ^c^ According to the size of the stem parts during extraction. ^d^ According to the extraction method. ^e^ According to the irrigation of the vine.

**Table 3:** Quantification of phenolic acids in white and red grape varieties (mg/kg DM).

Grape Variety	Vintage	Caftaric Acid	Coutaric Acid	Coumaric Acid	Gallic Acid	Caffeic Acid	Syringic Acid	Ferulic Acid
White Grape Varieties								
Aidani [29]	2009	71.6		0.9	171	1.4	3.5	1.1
Aidani [29]	2010	136		0.1	105	0.1	n.d.	n.d.
Arinto [48]	2018	220		40				
Asyrtiko [29]	2009	69.6		1.1	469	n.d.	1.8	n.d.
Asyrtiko [29]	2010	146		0.7	454	0.4	n.d.	n.d.
Athiri [29]	2009	5.1		0.6	122	0.5	0.4	n.d.
Athiri [29]	2010	6.1		0.7	146	0.5	0.5	0.2
Chasselas [39]	2015	1500 to 3600 ^b^			30 to 250 ^b^			
Fernao Pires [47]	-	100						
Fernao Pires [44]	2017	1710						
Fernao Pires [48]	2018	680			50			
Malvasia Fina [44]	2017	12,820						
Moscatel (Sanfins du Douro) [33]	2017	135						
Moscatel (Sanfins du Douro) [33]	2018	342						
Moscatel (Penajóia) [33]	2017	480						
Moscatel (Penajóia) [33]	2018	485						
Moscatel (Medrões) [33]	2017	203						
Moscatel (Medrões) [33]	2018	338						
Moscatel [44]	2017	5010						
Rabigato [47]	-	250						
Rabigato [44]	2017	2280						
Viosinho [47]	-	200						
Viosinho [44]	2017	1510						
**Red Grape Varieties**								
Amarela [47]	-	300						
Cabernet Sauvignon (irrigated) [28]	2000	20.3	11.7	8.1	6.9		14.7	
Cabernet Sauvignon (non irrigated) [28]	2000	30.8	2.7	1.4	n.d.		11.4	
Cabernet Sauvignon [39]	2015	900 to 1500 ^b^			80 to 260 ^b^			
Castelao [48]	2018	200			n.d.			
Mandilaria [29]	2009	57.9		0.9	286	5.3	1.4	0.7
Mandilaria [29]	2010	41.1		1.5	70.4	1.3	n.d.	0.6
Mavrotragano [29]	2009	166		4	182	9.2	1.2	1.5
Mavrotragano [29]	2010	78.4		1.1	90	2.8	0.3	n.d.
Mazuelo [38]	2016				43 to 310 ^c^			
Merlot [40]	-	40 ^a^	4.5 ^a^					
Merlot [39]	2015	800 to 1400 ^b^			80 to 300 ^b^			
Nerello Mascalese (Milo) [34]	2009				87.2			
Nerello Mascalese (Lingualossa) [34]	2009				71.9			
Nero d’Avola [34]	2009				49.8			
Sousao [47]	-	900						
Syrah (irrigated) [28]	2000	12.5	n.d.	n.d.	7.6		n.d.	
Syrah (non irrigated) [28]	2000	95.6	38	9	n.d.		n.d.	
Syrah [39]	2015	200 to 1600 ^b^			10 to 150 ^b^			
Syrah [48]	2018	400			40			
Tempranillo (irrigated) [28]	2000	31.5	16.3	n.d.	n.d.		n.d.	
Tempranillo (non irrigated) [28]	2000	66.9	32.8	0.9	n.d.		n.d.	
Tinta Barroca [47]	-	1100						
Tinta Roriz [48]	2018	430			n.d.			
Touriga Nacional [47]	-	500						
Touriga Nacional [48]	2018	980			n.d.			
Voidomato [29]	2009	274		2	195	8.6	2.4	n.d.
Voidomato [29]	2010	53.9		0.7	278	3.5	1.4	n.d.

^a^ Unit mg/kg FM. ^b^ According to the size of the stem parts during extraction. ^c^ According to the extraction method.

**Table 4:** Quantification of *trans*-resveratrol and ε-viniferin in white and red grape varieties (mg/kg DM).

Grape Variety	Vintage	*trans*-Resveratrol	*ε*-Viniferin
**White Grape Varieties**			
Aidani [29]	2009	74	167
Aidani [29]	2010	124	174
Arinto [48]	2018	80	70
Asyrtiko [29]	2010	178	253
Asyrtiko [29]	2009	87.6	223
Athiri [29]	2009	96	415
Athiri [29]	2010	115	499
Castealo [48]	2018	70	70
Chardonnay [52]	2010	n.d.	60.6
Chardonnay [52]	2012	42.2	25.7
Fernao Pires [53]	2013		1.91
Fernao Pires [44]	2017		900
Fernao Pires [48]	2018	90	80
Gewürztraminer [51]	-	393 ^a^	69 ^a^
Malvasia Fina [44]	2017		170
Moscatel [44]	2017		760
Moscatel (Medrões) [33]	2017		97
Moscatel (Medrões) [33]	2018		122
Moscatel (Penajóia) [33]	2017		75
Moscatel (Penajóia) [33]	2018		96
Moscatel (Sanfins du Douro) [33]	2017		23
Moscatel (Sanfins du Douro) [33]	2018		39
Moscato [51]	-	100 ^a^	56 ^a^
Palomino fino [52]	2010	traces	24.7
Palomino fino [52]	2012	n.d.	14.3
Pinot Gris [51]	-	159 ^a^	34 ^a^
Rabigato [53]	2013		29.9
Rabigato [44]	2017		310
Sauvignon [51]	-	95 ^a^	171 ^a^
Sauvignon blanc [52]	2010	n.d.	147.1
Tocai friulano [51]	-	82 ^a^	35 ^a^
Vijiriega [52]	2010	traces	48
Viosinho [53]	2013		26.1
Viosinho [44]	2017		830
**Red Grape Varieties**			
Cabernet franc [51]	-	238 ^a^	138 ^a^
Cabernet Sauvignon [52]	2012	n.d.	17.6
Granacha [52]	2010	traces	29.4
Mandilaria [29]	2009	266	476
Mandilaria [29]	2010	176	282
Marzemino [51]	-	31 ^a^	n.d.
Mavrotragano [29]	2009	87.6	258
Mavrotragano [29]	2010	96.5	235
Mazuelo [38]	2016	21 to 162 ^b^	91 to 310 ^b^
Merlot [51]	-	38 ^a^	54 ^a^
Merlot [52]	2012	n.d.	30.1
Nerello Mascalese (Lingualossa) [34]	2009	158.85	176.13
Nerello Mascalese (Milo) [34]	2009	102.63	114.75
Nero d’Avola [34]	2009	111.07	25.80
Petit Verdot [52]	2012	n.d.	20.5
Sousao [53]	2013		24.8
Syrah [48]	2018	60	70
Syrah [52]	2010	122.5	71.1
Syrah (treatment A) [52]	2010	135.4	52
Syrah (treatment A) [52]	2011	64	41.7
Syrah (treatment B) [52]	2011	139.1	65.1
Tempranillo 1 [52]	2010	79.8	60.5
Tempranillo 2 [52]	2010	87.8	80.6
Tempranillo [52]	2012	n.d.	28.3
Tinta Amarela [53]	2013		2.2
Tinta Baroca [53]	2013		10.8
Tinta Roriz [48]	2018	70	70
Tintilla de Rota [52]	2010	118.9	91.6
Tintilla de Rota [52]	2012	traces	39.2
Touriga Nacional [53]	2013		11.4
Touriga Nacional [48]	2018	140.00	110
*Vitis silvestris* 1 [52]	2010	49.9	59
*Vitis silvestris* 2 [52]	2010	traces	38.7
*Vitis silvestris* 3 [52]	2010	33	74.8
Voidomato [29]	2010	174	414
Voidomato [29]	2009	92.9	217

^a^ Unit mg/kg FM. ^b^ According to the extraction method.

**Table 5:** Flavonol content quantified in different grape stem extracts (mg/kg DM).

Grape Variety	Vintage	Quercetin-3-O-Galactoside	Quercetin-3-O-Glucoside	Quercetin-3-O-Rhamnoside	Quercetin-3-O-Glucuronide	Quercetin-3-O-Rutinoside	Quercetin	Kaempferol	Kaempferol-3-O-Rutinoside	Kaempferol 3-O-Glucoside
**White Grape Varieties**										
Aidani [29]	2009	87.2	57.7	17.3			9.4	0.5		
Aidani [29]	2010	197	71.5	19.3			7.3	1		
Arinto [48]	2018					150				
Asyrtiko [29]	2009	193	65.1	4.6			21	1.3		
Asyrtiko [29]	2010	305	137	24.1			5.6	0.8		
Athiri [29]	2009	142	50.9	15.8			7.7	1.4		
Athiri [29]	2010	170	61.1	19			9.2	1.6		
Chasselas [39]	2015					600 to 3000 ^b^				
Fernao Pires [47]	-				400	70.0			60	50
Fernao Pires [44]	2017				40,270	140			140	40
Fernao Pires [48]	2018					440				
Malvasia Fina [47]	2017				73,790	190			400	20
Moscatel (Sanfins du Douro) [33]	2017		211			22			5	19
Moscatel (Sanfins du Douro) [33]	2018		285			117			8	21
Moscatel (Penajóia) [33]	2017		387			45			10	29
Moscatel (Penajóia) [33]	2018		445			187			12	30
Moscatel (Medrões) [33]	2017		374			36			7	27
Moscatel (Medrões) [33]	2018		423			104			10	29
Moscatel [44]	2017				29,270	230			140	40
Viosinho [44]	2017				34,630	50			100	40
Viosinho [47]					800	80.0			30	75
**Red Grape Varieties**										
Amarela [47]	-				600	50.0			55	33
Cabernet Sauvignon [39]	2015					500 to 800 ^b^				
Castelao [48]	2018					240				
Mandilaria [29]	2009	127	54.1	6.7			12.7	4.4		
Mandilaria [29]	2010	243	130	4.2			10.3	0.7		
Mavrotragano [29]	2009	223	86.5	17.5			2	0.7		
Mavrotragano [29]	2010	149	70.1	8.4			9.5	1.8		
Mazuelo [38]	2016		96 to 485 ^c^				8 to 38 ^c^			
Merlot [39]	2015					200 to 1000 ^b^				
Merlot [40]	-		18.0 ^a^		200.0 ^a^				traces ^a^	
Nerello Mascalese (Milo) [34]	2009		36.4		70.7					
Nerello Mascalese (Lingualossa) [34]	2009		152.9		229.5					
Nero d’Avola [34]	2009		65.7		161.3					
Rabigato [47]	-		350.0			50.0			25	25
Rabigato [44]	2017				37,560	150			40	20
Sousao [47]	-				1380	120.0			75	25
Syrah [39]	2015					50 to 600 ^b^				
Syrah [48]	2018					410				
Tinta Barroca [47]					140	120			150	30
Tinta Roriz [48]	2018					370				
Touriga Nacional [47]					700	25			80	20
Touriga Nacional [48]	2018					440				
Voidomato [29]	2009	205	65.5	15.3			13.7	n.d.		
Voidomato [29]	2010	126	61.4	23.8			19.6	2.3		

^a^ Unit mg/kg FM. ^b^ According to the size of the stem parts during extraction. ^c^ According to the extraction method.

**Table 6:** Identification of flavan-3-ols and proanthocyanidins in grape stem extracts (ESI).

Compound	[M-H]− (m/z)	[M-H]+ (m/z)	MS2 (m/z)
(+)-catechin [41]	289		245; 205; 179; 203; 227; 165; 161
(+)-catechin [37,41]		291	123; 139; 165; 273; 151; 147; 249
(−)-epicatechin [29,40,41,47,55]		291	245; 205; 179; 203; 231; 271; 161
(−)-epicatechin [41]	289		123; 139; 165; 151; 273; 147; 231
(epi)catechin gallate [29,47,55]	441		331; 289; 169
Catechin gallate [41]	441		289; 395; 169; 331; 245; 193; 405
Procyanidin dimer A [47]	575		573; 477; 441
B1 Ec-(4β→8)-Cat [41]	577		425; 407; 289; 451; 245; 287
B1 Ec-(4β→8)-Cat [41]		579	427; 409; 291; 301; 247; 289; 287
B2 Ec-(4β→8)-Ec [41]	577		425; 407; 287; 289; 451; 559; 299
Procyanidin dimer B [47]	577		559; 425; 407; 287
Procyanidin dimer [36]	577		289; 425; 407; 451; 559
(epi)gallocatechin-(epi)catechin dimer [47]	593		575; 531; 425; 423; 273
Galloylated flavanol dimer (epi)catechin-(epi)catechin gallate [37,49]		731	579; 291; 139
Procyanidin dimer gallate	729		711; 577; 559; 451; 407; 289
Procyanidin dimer gallate [36]	729		577; 559; 451; 407; 425; 289
Prodelphinidine gallate [36]	745		593; 405; 575
Flavanol dimer [37]		579	601
Flavanol trimer [49]		867	579; 427
Procyanidin trimer [36]	865		695; 577; 739; 451
Prodelphinidin trimer [36]	881		695; 577; 755; 407; 303
Procyanidin trimer Gallate [36]	1017		729; 407
Procyanidin tetramer [36]	1153		865
Procyanidin tetramer [36]	1169		881; 999; 1043; 729
Procyanidin pentamer [M-H]^2−^ [36]	720		635; 577; 521; 407

**Table 7:** Quantification of different flavan-3-ols and proanthocyanidins in grape stem extracts.

Grape Variety	Vintage	Cat	Ec	EcG	B1	B2	B3	B4
**Unit: mg/g FM**								
Castelao Frances [56]	1998	1.3	0.7		3.5	0.4	0.2	1
Merlot [40]	-	60	traces					
Tinta Miuda [57]	1996	64.4	2.2		128.2	3.4	27.1	3.1
Touriga Francesa [56]	1998	2	0.5		5.8	1.2	1.2	0.4
Viosinho [56]	1998	1.5	0.6		1.2	0.4	0.1	0.1
**Unit: mg/kg DM**								
Aidani [29]	2009	699	51.6	77.0		48.8	383	
Aidani [29]	2010	737	58	34.2		36	215	
Arinto [48]	2018	660	30					
Asyrtiko [29]	2009	1089	18.2	59.1		36.2	454	
Asyrtiko [29]	2010	1858	27.9	86.0		165	646	
Athiri [29]	2009	385	n.d.	53.9		55.2	161	
Athiri [29]	2010	462	12.3	64.7		66.2	193	
Cabernet Sauvignon [39]	2015	500 to 800						
Cabernet Sauvignon [31]	2009	493	31		564	21	120	n.d.
Cabernet Sauvignon (irrigated) [28]	2000	n.d.	7.6					
Cabernet Sauvignon (non-irrigated) [28]	2000	368.8	n.d.					
Callet [31]	2009	453	16		454	20	156	n.d.
Castelao [48]	2018	440	40					
Chardonnay [31]	2009	314	12		255	15	56	n.d.
Chasselas [39]	2015	600 to 3000						
Fernao Pires [48]	2018	1270	170					
Macabeu [31]	2009	93	0.5		133	11	45	n.d.
Mandilaria [29]	2009	1261	70.9	108.0		96.6	482	
Mandilaria [29]	2010	1691	94.6	71.3		46.2	993	
Manto Negro [31]	2009	122	06		246	11	41	n.d.
Mavrotragano [29]	2009	1077	79.8	130.0		108	587	
Mavrotragano [29]	2010	1027	64.4	88.0		44.3	243	
Mazuelo [38]	2016	225 to 710						
Merlot [31]	2009	575	24		868	22	132	n.d.
Merlot [39]	2015	200 to 1000						
Nerello Mascalese (Milo) [34]	2009	3611.0			1370.2			
Nerello Mascalese (Lingualossa) [34]	2009	2066.3			793.3			
Nero d’Avola [34]	2009	1562.7			1771.4			
Parellada [31]	2009	1339	58		1877	48	222	n.d.
Premsal blanc [31]	2009	740	40		1218	40	104	n.d.
Syrah [31]	2009	1146	24		1320	traces	208	
Syrah [39]	2015	50 to 600						
Syrah [48]	2018	1330	110					
Syrah (irrigated) [28]	2000	n.d.	24.1					
Syrah (non-irrigated) [28]	2000	n.d.	n.d.					
Tempranillo [31]	2009	1269	111		1958	94	232	n.d.
Tempranillo [42]	-	7640						
Tempranillo (irrigated) [28]	2000	674.4	338.5					
Tempranillo (non-irrigated) [28]	2000	280.8	n.d.					
Tinta Roriz [48]	2018	1620	90					
Touriga Nacional [48]	2018	2030	180					
Voidomato [29]	2009	795	189	95.3		n.d.	349	
Voidomato [29]	2010	712	n.d.	64.9		n.d.	138	

Cat = catechin; Ec = epicatechin; EcG = epicatechin gallate; B1, 2, 3 and 4 = proanthocyanidins dimers.

**Table 8:** Total proanthocyanidin content of grape stem extracts (mg/g DM).

Grape Variety	Total Proanthocyanidin
**Method: Bate-Smith Reaction**	
Cabernet Sauvignon [31]	124.9
Callet [31]	202.3
Chardonnay [31]	79.1
Macabeu [31]	108.8
Manto Negro [31]	165.3
Merlot [31]	84.0
Parellada [31]	165.2
Premsal blanc [31]	181.4
Syrah [31]	161.4
Tempranillo [31]	147.3
Manto Negro [23]	103
Premsal blanc [21]	79.0
Roditis [35]	55.5 to 255.7 ^b,d^
**Method: LCMS/MS Quantification**	
Amarela [47]	39
Fernao Pires [47]	35
Rabigato [47]	27
Sousao [47]	45
Tinta Barroca [47]	45
Touriga Nacional [47]	37
Viosinho [47]	27
**Method: Vanillin Assay**	
Castelao Frances [56]	53.7 ^a^
Manto Negro [45]	between 217 and 270 ^c^
Premsal blanc [45]	between 126 and 162 ^c^
Tinta Miuda [57]	2.2 ^a^
Touriga Francesa [56]	52.8 ^a^
Viosinho [56]	37.8 ^a^
**Methyl Cellulose Precipitation**	
Tempranillo [42]	24.29

^a^ Unit mg/g FM. ^b^ mg CyE/100g DM. ^c^ mg CAE/g DM. ^d^ According to the extraction method.

**Table 9:** Mean degree of polymerization (mDP) and structural composition of stem polymeric proanthocyanidins.

Grape Variety		mDP	General Composition				Terminal Units				Extension Units			
			% Cat	% Ec	% EcG	% EgC	% Cat	% Ec	% EcG	% EgC	% Cat	% Ec	% EcG	% EgC
Cabernet sauvignon [31]	**Phloroglucinolysis Method**	5.9	25	74	1.0		97	tr	3		11	89		
Callet [31]		4.7	29	70	1.0		89	7	4		12	88		
Chardonnay [31]		4.6	28	71	1.0		89	6	5		11	89		
Macabeu [31]		6.2	24	75	1.0		83	11	6		13	87		
Manto Negro [31]		5.8	26	73	1.0		97	tr	3		11	89		
Merlot [31]		6.0	25	75	0.0		97	tr	3		10	90		
Parellada [31]		5.0	27	72	1.0		95	2	3		10	90		
Premsal blanc [31]		8.5	25	74	1.0		95	tr	4		16	84		
Syrah [31]		6.1	22	77	1.0		97	tr	3		7	93		
Tempranillo [31]		6.9	20	79	1.0		95	tr	5		8	92		
Stems *Vitis vinifera* sp. [55]	**Thiolysis Method**	5	16.8	55.3	17.1	10.5	84.2	11.3	4.5	n.d.	6.5	62.3	19.1	12.2
Commercial stem powder [55]		6.6	23.7	59.3	8.0	8.9	100.0	n.d.	n.d.	n.d.	13.5	67.3	9.1	10.1
Chardonnay [40]		9.1	14	69.4	15.7	0.8								
Clairette [40]		7.7	17.3	68.4	13.4	0.9								
Merlot [40]		9.2	14.4	67.7	15.6	2.4	8.6	1.8	0.6		5.8	65.8	15.0	2.4
Négrette [40]		10.2	11.7	61.7	21.1	5.4								
Pinot [40]		8.2	15.3	65.1	18.1	1.5								
Tannat [40]		8.7	13	65.5	19.8	1.7								

mDP = Mean degree of polymerization; Cat = catechin; Ec = epicatechin; EcG = epicatechin gallate; Egc = epigallocatechin.

**Table 10:** Antioxidant activity of grape stem extracts (ABTS and FRAP methods).

Grape Variety	ABTS	FRAP
**Unit: mg Trolox/g DM**		
Arinto [48]	87.6	87.6
Cabernet Sauvignon [31]	168.9	114.8
Callet [31]	253.2	170.1
Castelao [48]	115.1	140.2
Chardonnay [31]	99.7	65.4
Fernao Pires [44]	150.2	
Fernao Pires [48]	172.7	247.8
Macabeu [31]	131.7	85.5
Malvasia fina [44]	275.3	
Manto Negro [31]	198.2	134.6
Merlot [31]	109.8	76.6
Moscatel [44]	300.3	
Parellada [31]	223.4	159.1
Premsal blanc [31]	218.5	169.1
Rabigato [44]	250.3	
Syrah [31]	203.1	155.3
Syrah [48]	147.7	212.7
Tempranillo [31]	186.8	127.4
Tinta Roriz [48]	175.2	235.3
Touriga Nacional [48]	210.2	257.8
Viosinho [44]	200.2	
**Unit: mM Trolox/100 g DM**		
Amarela [47]	57	37
Fernao Pires [47]	31	25
Mazuelo [38]	8 to 30 ^a^	4 to16 ^a^
Moscatel (Sanfins du Douro) 2017 [33]	38	33
Moscatel (Sanfins du Douro) 2018 [33]	67	74
Moscatel (Penajóia) 2017 [33]	73	84
Moscatel (Penajóia) 2018 [33]	73	85
Moscatel (Medrões) 2017 [33]	41	41
Moscatel (Medrões) 2018 [33]	69	75
Rabigato [47]	32	20
Sousao [47]	70	46
Tinta Barroca [47]	59	40
Touriga Nacional [47]	50	30
Viosinho [47]	40	24

^a^ According to the extraction method.

**Table 11:** Identification of aromatic compounds in grape stem extracts.

Grape Variety	Compound	Retention Index			Odor Description
		DB-WAX	Ultra-1	DB-5	
Cabernet Sauvignon and Chardonnay [73]	hexanal	1099	800		green
	(E)-2- hexanal	1200	844		green
	(Z)-1,5-octandien-3-one	1346	963		geranium-like, metallic green
	2-methoxy-3-isopropylpyrazine	1394	1092		grassy, earthy
	unknown	1484	-		cucumber-like
	2-methoxy-3-isobutylpyrazine	1500	1211		herbaceous, earthy
	(E,Z)-2,6-nonadienal	1561	1150		cucumber-like
	dodecanal	1737	1402		citrus skin -like
Syrah [76]	3-isobutyl-2-methoxypyrazine	1530		1184	green pepper
	γ-octalactone	1877		1276	sweet, almond, coconut
	*trans*-4,5-epoxy-E-2-decenal	2011		1381	metallic
	furaneol (2.5-dimethyl-4-3(2H)-furanone)	2036		1073	caramel, red fruit jam aroma
	eugenol (4-allyl-2-methoxyphenol)	2171		1365	clove
	sotolon (3-hydroxy-4.5-dimethylfuran-2(5H)-one	2198		1105	curry
	vanillin	1564		1407	vanilla

**Table 12:** pH and acidic composition of wine made with and without stem addition (for each study, different letters indicate significant statistical differences).

Grape Variety	Modality	Maceration Time (Days)	pH	Titrable Acidity (g/L)	Total Acidity (g/L Tartaric Acid)	Volatile Acidity (g/L Acetic Acid)	Tartaric Acid (g/L)	Malic Acid (g/L)	Lactic Acid (g/L)	Acetic Acid (mg/L)	Phosphoric Acid (mg/L)	Citric Acid (mg/L)	Succinic Acid (g/L)	Shikimic Acid (mg/L)
Cabernet sauvignon [3]	no stem	7	3.78				1.446b	4.220	0.322	161	795a	571a	1.576	
	stem addition		3.85				1.308a	4.303	0.304	165	948b	633b	1.486	
Cabernet sauvignon [12]	no stem	15	3.23a	5.5b										
	stem addition		3.45b	4.7a										
Castelao [79]	no stem	7	3.19		7.25	0.48	2.7b	1.700	0.0					
	stem addition		3.22		7.15	0.47	2.6a	1.700	0.0					
	no stem	21	3.19a		7.25	0.48	2.7b	1.700	0.0a					
	stem addition		3.28b		6.55	0.49	2.5a	0.900	0.300b					
Merlot [3]	no stem	7	3.54											
	stem addition		3.54											
Muscat bailey A [3]	no stem	7	3.83a				1.105b	3.787	0.343	276	669a	729	1.228b	
	stem addition		3.95b				1.011a	3.790	0.382	247	926b	767	1.032a	
Pinot Noir [3]	no stem addition	7	3.60a											
	stem addition		3.69b											
Pinot Noir 2014 [84]	no stem	10	3.74											
	stem addition		3.74											
Pinot Noir 2015 [84]	no stem	10	3.66bc	4.03b		0.90b		0.760	1.31					
	stem addition		3.71c	3.86a		0.94b		0.660	1.30					
	20% whole cluster		3.63ab	3.96a		0.85a		0.810	1.31					
Pinot Noir 2016 [83]	no stem addition	10	3.36b	6.4b		0.77b		0.05	0.86ab					
	50% whole cluster		3.55a	6.8a		0.81b		0.05	0.81ab					
	100% whole cluster		3.53a	6.8a		1.11a		0.06	0.79b					
	dried stems		3.52a	6.5ab		0.85b		0.06	0.87a					
Pinot Noir 2017 [83]	no stem addition	10	3.31c	7.1		0.79b		0.04	0.58b					
	50% whole cluster		3.42bc	7.2		0.95ab		0.04	0.66ab					
	100% whole cluster		3.60a	7.2		1.12a		0.04	0.76a					
	dried stems		3.51ab	7.1		0.91ab		0.06	0.72a					
Primitivo [81]	no stem	10	3.91		6.15	0.75	1.60	1.73	0.04			1.14	1.28	18.4a
	25% whole cluster		3.84		6.38	0.75	1.53	2.4	0.05			0.96	1.23	23.7b
	50% whole cluster		3.90		6.3	0.66a	1.69	1.7	0.06			0.97	1.33	22.4b
Tinta Miuda [80]	no stem	6	3.0		8.6	0.8								
	stem addition		3.0		8.5	0.7								

**Table 13:** Mineral composition of wines made with and without stem addition (mg/L) (for each study, different letters indicate significant statistical differences).

Grape Variety	Modality	K	P	Ca	Mg	Na	Mn	Fe	Cu	Zn
Cabernet Sauvignon [3]	no stem addition	1454a	198a	62a	71	4.4	0.6	5.8	0.5a	0.3
	stem addition ^a^	1927b	277b	69b	73	4.8	0.8	5.0	0.9b	0.3
Muscat Bailey A [3]	no stem addition	2046a	208a	74a	55a	4.7	0.8	5.1	0.9	0.5a
	stem addition ^a^	2476b	389b	90b	60b	4.7	0.9	7.0	0.8	0.8b
Tinta Miuda [80]	no stem addition	1065.8a		79.2a	88.0a	12.4a		2.4	0.1	
	stem addition ^b^	1088.6b		104.0b	96.0b	20.0b		2.0	0.1	

Stems were left in contact with the must for: ^a^ 7 days; ^b^ 6 days.

**Table 14:** Ethanol content in wine made with and without stem addition (for each study, different letters indicate significant statistical differences).

Grape Variety	Modality	Maceration time (Days)	Alcohol (% *v*/*v*)
Cabernet sauvignon [12]	no stem addition	15	≈13b
	stem addition		≈12.6a
Castelao [79]	no stem addition	7	13.3
	stem addition		13.3
	no stem addition	21	13.3
	stem addition		13.2
Pinot Noir 2014 [84]	no stem addition	7	13.03b
	stem addition		12.75a
Pinot Noir 2015 [84]	no stem addition	10	15.16b
	stem addition		15.03b
	20% whole cluster		14.31a
Pinot Noir 2016 [83]	no stem addition	10	13.07
	50% whole cluster		13.24
	100% whole cluster		13.02
	dried stems		13.48
Pinot Noir 2017 [83]	no stem addition	10	14.54ab
	50% whole cluster		13.90b
	100% whole cluster		14.24ab
	dried stems		14.68a
Primitivo [81]	no stem addition	10	19.67b
	25% whole cluster		19.38c
	50% whole cluster		20.05a
Tinta Miuda [80]	no stem addition	6	8.4b
	stem addition		7.7a

**Table 15:** Total polyphenol compounds in wine made with and without stem addition (for each study, different letters indicate significant statistical differences).

Grape Variety	Modality	Maceration Time (days)	Total Phenolic Compounds (FI) (mg GAE/L)	Total Polyphenol Index (TPI)
Cabernet sauvignon [12]	no stem addition	15		42.0a
	stem addition			48.2b
Cabernet sauvignon [3]	no stem addition	7	1769a	
	stem addition		2160b	
Castelao [79]	no stem addition	7		46.2
	stem addition			50.0
	no stem addition	7		46.2
	stem addition	21		49.0
Merlot [3]	no stem addition	7	1483a	
	stem addition		1923b	
Muscat bailey A [3]	no stem addition	7	1334a	
	stem addition		1671b	
Pinot Noir [3]	no stem addition	7	1013	
	stem addition		1100	
Primitivo [81]	no stem addition	10	2685a	
	25% whole cluster		3127b	
	50% whole cluster		3164b	
Tinta Miuda [80]	no stem addition	6		26.47a
	stem addition			32.19b

**Table 16:** Impact of stems on phenolic acids composition of the wine (mg/L) (for each study, different letters indicate significant statistical differences).

Grape Variety	Modality	Maceration Time (Days)	Gallic Acid	Syringic Acid	Caftaric Acid	2-S-Gluthationyl Caftaric Acid	*trans* p-Coutaric Acid	*cis* p-Couratic Acid	Fertaric Acid	Caffeic Acid	*Trans* p-Coumaric Acid	Ferulic Acid
Palomino fino [85]	100% whole cluster	9	2.40	1.44a	36.98	7.38	9.08	3.49	0.65	5.09a	0.38	0.32
	75% whole cluster	9	10.47	1.76	40.95a	10.12	9.49b	3.62	0.65	2.82b	0.41b	0.62
	50% whole cluster	9	6.08	1.20	37.17b	8.36	9.24a	3.49a	0.64	4.27	0.27b	0.35
	25% whole cluster	9	3.29	1.82b	38.57	8.53	10.1	4.27b	0.86	5.00	0.56a	0.41
Cabernet sauvignon [12]	no stem addition	15			18.38		1.03	0.62	0.45	2.12		
	stem addition				20.24		1.03	0.72	0.50	2.20		

**Table 17:** Impact of stems during winemaking on total proanthocyanidin content (for each study, different letters indicate significant statistical differences).

Grape Variety	Vintage	Modality	Maceration Time (days)	Total Proanthocyanidin (mg/L)
**Method: Bate-Smith Reaction**				
Cabernet [30]	1966	no stem addition	4	1700
		stem addition		2100
Cabernet [30]	1966	no stem addition	20	4200
		stem addition		4500
Cabernet [30]	1967	no stem addition	n.d.	1700
		stem addition		2000
Malbec [30]	1966	no stem addition	4	2400
		stem addition		3500
		no stem addition	8	3200
		stem addition		3900
		no stem addition	14	3500
		stem addition		4500
		no stem addition	30	3700
		stem addition		4700
Merlot [30]	1966	no stem addition	8	2500
		stem addition		4000
**Method: Vanillin Assay**				
Primitivo [81]	2012	no stem addition	10	1744a
		25% whole cluster		2180b
		50% whole cluster		2275b
**Method: Precipitation Methods**				
Cabernet sauvignon ^1^ [12]	2013	no stem addition	15	403a
		stem addition		778b
Pinot Noir ^2^ [84]	2014	no stem addition	10	370
		20% whole cluster		350
Pinot Noir ^2^ [84]	2015	no stem addition	10	540b
		stem addition		860c
		20% whole cluster		440a
Pinot Noir ^2^ [83]	2016	no stem addition	10	100a
		50% whole cluster		210b
		100% whole cluster		320c
		dried stems		325c
Pinot Noir ^2^ [83]	2017	no stem addition	10	112a
		50% whole cluster		175a
		100% whole cluster		270c
		dried stems		275c

^1^ Methyl cellulose precipitation. ^2^ Protein precipitation (mg/L catechin equivalent (CE).

**Table 18:** Impact of stems during winemaking on flavan-3-ol monomeric and polymeric composition.

Grape Variety	Vintage	Modality	Maceration Time (Days)	Cat	Ec	Gc	EcG	Egc	EgcG	B1	B2	B3	B4
Cabernet sauvignon [12]	2013	stem addition	15	++	0	++		++					
Castelao [79]	2000	stem addition	7	+	0					+	0	0	0
		stem addition	21	++	++					0	0	0	0
Primitivo [81]	2012	25% whole cluster	10	+	+	−	0	+	+	+	+	+	++
		50% whole cluster	10	++	++	−−	+	+	+	++	+	+++	++
Tinta Miuda [57]	1996	stem addition	21	++	++					0	0	0	0
Tinta Miuda [80]	1998	stem addition	6	+++	−					+++	0	+++	0

Cat = catechin; Ec = epicatechin; Gc = gallocatechin; EcG = epicatechin gallate; Egc = Epigallocatechin; EgcG = Epigallocatechine gallate; B1, 2, 3 and 4 = proanthocyanidins dimers; % of variation: 0–50 (+/−); 50–100 (++/−−); 100–250 (+++/−−−); 250–500 (++++/−−−−); >500(+++++/−−−−−).

**Table 19:** Impact of stems during winemaking on total anthocyanin content (for each study, different letters indicate significant statistical differences).

Grape Variety	Vintage	Modality	Maceration Time (Days)	Total Anthocyanin (mg/L)
**Method: pH Variation—HCl**				
Primitivo [81]	2012	no stem addition	10	401 ^1^ a
		25% whole cluster		374 ^1^ b
		50% whole cluster		368 ^1^ b
Cabernet sauvignon [12]	2013	no stem addition	15	474.5 ^3^ b
		stem addition		426.4 ^3^ a
**Method: SO_2_ Bleaching**				
Cabernet [30]	1966	no stem addition	4	800 ^2^
		stem addition		690 ^2^
Cabernet [30]	1966	no stem addition	20	800 ^2^
		stem addition		690 ^2^
Cabernet [30]	1967	no stem addition	n.d.	710 ^2^
		stem addition		700 ^2^
Castelao [79]	2000	no stem addition	7	283 ^3^
		stem addition		261 ^3^
		no stem addition	21	283 ^3^ b
		stem addition		221 ^3^ a
Malbec [30]	1966	no stem addition	4	630 ^2^
		stem addition		570 ^2^
		no stem addition	8	610 ^2^
		stem addition		500 ^2^
		no stem addition	14	600 ^2^
		stem addition		540 ^2^
		no stem addition	30	390 ^2^
		stem addition		320 ^2^
Merlot [30]	1966	no stem addition	8	540^2^
		stem addition		580 ^2^
Pinot Noir [84]	2014	no stem addition	10	270 ^3^
		20% whole cluster		260 ^3^
Pinot Noir [84]	2015	no stem addition	10	250 ^3^
		stem addition		270 ^3^
		no stem addition	10	250 ^3^
		20% whole cluster		280 ^3^
Pinot Noir [83]	2016	no stem addition	10	251 ^3^
		50% whole cluster		250 ^3^
		100% whole cluster		251 ^3^
		dried stems		251 ^3^
Pinot Noir [83]	2017	no stem addition	10	150 ^3^
		50% whole cluster		140 ^3^
		100% whole cluster		135 ^3^
		dried stems		150 ^3^
Tinta Miuda [80]	1998	no stem addition	6	148.77 ^3^ b
		stem addition		129.72 ^3^ a

^1^ mg/L malvidin chloride. ^2^ mg/L unspecified reference. ^3^ mg/L malvidin-3-O-glucoside.

**Table 20:** Impact of stems during winemaking on individual anthocyanin content (mg/L) (for each study, different letters indicate significant statistical differences).

Grape Variety	Vintage	Modality	Maceration Time (Days)	Delphinidin-3-O-G	Cyanidin-3-O-G	Petunidin-3-O-G	Peonidin-3-O-G	Malvindin-3-O-G	Acetylated Anthocyanins	P-Coumarylated Anthocyanins
**Reference Standard: Malvidin Chloride**										
Primitivo [81]	2012	no stem addition	10	5.67a	0.77a	15.31a	8.72a	181.37a	19.6b	26.44a
		25% whole cluster		4.42b	0.66b	12.6b	7.51b	150.50b	21.65a	22.98b
		50% whole cluster		4.3b	0.54c	12.25b	7.31b	149.21b	18.27b	22.36b
**Reference Standard: Malvidin-3-O-Glucoside**										
Cabernet sauvignon [12]	2013	no stem addition	15						27.2	11.6b
		stem addition							27.0	8.9a
Castelao [79]	2000	no stem addition	7	10.8b	1.6b	13.5b	19.1b	115.9b	n.d.	16.6b
		stem addition		9.1a	1.4a	10.9a	15.6a	91.9a	n.d.	15.1a
		no stem addition	21	10.8b	1.6b	13.5b	19.1b	115.9b	n.d.	16.6
		stem addition		8.1a	1.2a	9.3a	13.2a	80.2a	n.d.	12.3
Pinot Noir [84]	2014	no stem addition	10	4	n.d.	9	28	168		
		20% whole cluster		4	n.d.	9	28	160		
Pinot Noir [84]	2015	no stem addition	10	4	3	8	24	96		
		stem addition		4	3	8	25	96		
		20% whole cluster		4	3	8	25	112		
Tinta Miuda [80]	1998	no stem addition	6	5.73b	1.89b	6.59b	17.51b	62.96b	12.77	15.2
		stem addition		5.19a	1.86a	5.93a	13.94a	51.52a	11.81	13.15

**Table 21:** Impact of stems on color intensity and hue of wine (for each study, different letters indicate significant statistical differences).

Grape Variety	Vintage	Modality	Maceration Time (Days)	Color Intensity A_420_ + A_520_ + A_620_	Hue A_420_/A_520_
Cabernet [30]	1966	no stem addition	4	1.51	0.42
		stem addition		1.11	0.55
Cabernet [30]	1966	no stem addition	20	1.39	0.55
		stem addition		1.24	0.43
Cabernet [30]	1967	no stem addition	nd	1.35	0.41
		stem addition		1.21	0.51
Cabernet sauvignon [3]	1996	no stem addition	7		0.505a
		stem addition			0.592b
Cabernet sauvignon [12]	2013	no stem addition	15	9.7b	0.529a
		stem addition		8.1a	0.583b
Malbec [30]	1966	no stem addition	4	1.52	0.52
		stem addition		1.22	0.56
		no stem addition	8	1.62	0.56
		stem addition		1.22	0.57
		no stem addition	14	1.36	0.51
		stem addition		1.33	0.59
		no stem addition	30	1.2b	0.67
		stem addition		1.19	0.67
Merlot [30]	1966	no stem addition	8	1.41	0.55
		stem addition		1.19	0.54
Merlot [3]	1996	no stem addition	7		0.630a
		stem addition			0.717b
Muscat Bailey A [3]	1996	no stem addition	7		0.758a
		stem addition			0.866b
Pinot Noir [3]	1996	no stem addition	7		1.020a
		stem addition			1.133b
Pinot Noir [84]	2014	no stem addition	10	0.39	
		20% whole cluster		0.36	
Pinot Noir [84]	2015	no stem addition	10	0.6	
		stem addition		0.64	
		no stem addition	10	0.6	
		20% whole cluster		0.56	
Pinot Noir [83]	2016	no stem addition	10	0.5	
		50% whole cluster		0.57	
		100% whole cluster		0.59	
		dried stems		0.65	
Pinot Noir [83]	2017	no stem addition	10	0.58	
		50% whole cluster		0.52	
		100% whole cluster		0.57	
		dried stems		0.6	
Primitivo [81]	2012	no stem addition	10	11.97a	0.72b
		25% whole cluster		15.9b	0.67a
		no stem addition	10	11.97a	0.72b
		50% whole cluster		15.2b	0.70a

**Table 22:** Impact of stems on methoxypyrazine composition in wine samples.

Grape Variety	Modality	Methoxypyrazine Compounds		
		IPMP (ng/L)	SBMP (ng/L)	IBMP (ng/L)
Cabernet Sauvignon [73]	No stem addition	n.d.	n.d.	25.3
	Stem addition	2.7	2.8	33.8
Chardonnay [73]	No stem addition	n.d.	n.d.	11.6
	Stem addition	2.5	2.0	18.0
Sauvignon Blanc [82]	No stem addition	0.85	n.d.	4.8
	Stem addition	3.6	0.8	14.1

n.d.: not detected; IPMP = 2-methoxy-3-isopropylpyrazine; SBMP = 2-methoxy-3-sec-butylpyrazine; IBMP = 2-methoxy-3-isobutylpyrazine.

**Table 23:** Main effect of the use of stems or whole clusters on wines from an oenological point of view.

Parameter	Variation	Percentage of Change Compared to Fully Destemmed Wines	References
pH	(+)	1 to 9	[3,12,79,83,84]
	0		[3,79,80,81,84]
Titrable acitidy	(−)	2 to 15	[12,83,84]
	0		[79,83,84]
Volatile acidity	(+)	4 to 44	[83,84]
	(−)	6 to 12	[81,84]
	0		[79,83,84]
Potassium (K)	(+)	2 to 33	[3,80]
Ethanol content	(+)	1 to 3	[81,83]
	(−)	1 to 8	[12,80,81,83,84]
	0		[79,83]
Total polyphenolic content	(+)	14 to 30	[3,12,80,81]
	0		[3,79]
Total proanthocyanidin content	(+)	7 to 225	[12,30,81,84]
	(−)	19	[83,84]
	0		[84]
Total anthocyanin content	(+)	7 to 12	[30,84]
	(−)	1 to 22	[12,30,79,80,81,83,84]
	0		[81,83]
Color Intensity	(+)	7 to 33	[81]
	(−)	1 to 26	[12,30]
	0		[83,84]
Color hue	(+)	10 to 17	[3,12]
	(−)	3 to 7	[81]
	0		[30]
Aroma and volatile compounds	(+)	1-Hexanol, IPMP, SBMP, IBMP	[73,79,82,84,85]
Taste	(+)	astringency and bitterness	[12,83,84]
	(+)	complexity and freshness	[83]

## Abbreviations

ABTS	2,2-azinobis(3-ethylbenzthiazoline-6-sulphonic acid)
B1 (2, 3, 4 etc.)	Procyanidin dimer B1 (2, 3, 4 etc.)
CAE	Catechin equivalent
Cat	Catechin
CUPRAC	Cupric reducing antioxidant capacity
CyE	Cyanidin equivalent
DM	Dry matter
DMACA	4-(N,N′-dimethylamino)cinnamaldehyde
DPPH	1,1-diphenyl-2-picrylhydrazine
Ec	Epicatechin
EcG	Epicatechin gallate
Egc	Epigallocatechin
EgcG	Epigallocatechin gallate
ESI	Electron spray I
FI	Folin Index
FRAP	Ferric reducing antioxidant power
FW	Fresh weight
GAE	Gallic acid equivalent
Gc	Gallocatechin
GC—MS	Gas Chromatography—mass spectrometry
HPLC	High pressure liquid chromatography
IBMP	2-methoxy-3-isobutylpyrazine
ICP	Inductive coupled plasma
IPMP	2-methoxy-3-isopropylpyrazine
LCMS	Liquid chromatography—mass spectrometry
MC	Methyl cellulose
mDP	Mean degree of polymerization
ORAC	Oxygen radical absorbance capacity
OZ	Odorant zone
SBMP	2-methoxy-3-sec-butylpyrazine
TPI	Total polyphenolic index
